# The longitudinal biochemical profiling of TBI in a drop weight model of TBI

**DOI:** 10.1038/s41598-023-48539-x

**Published:** 2023-12-14

**Authors:** Ali Yilmaz, Sigal Liraz-Zaltsman, Esther Shohami, Juozas Gordevičius, Ieva Kerševičiūtė, Eric Sherman, Ray O. Bahado-Singh, Stewart F. Graham

**Affiliations:** 1 Metabolomics Department, Beaumont Research Institute, Beaumont Health, Royal Oak, MI 48073 USA; 2 Oakland University-William Beaumont School of Medicine, Rochester, MI 48073 USA; 3 Department of Pharmacology, The Institute for Drug Research, The Hebrew University of Jerusalem, Jerusalem, Israel; 4 The Joseph Sagol Neuroscience Center, Sheba Medical Center, Ramat-Gan, Israel; 5 Department of Sports Therapy, Institute for Health and Medical Professions, Ono Academic College, Qiryat Ono, Israel; 6 VUGENE LLC, 625 EKenmoor Avenue Southeast, Suite 301, PMB 96578, Grand Rapids, MI 49546 USA; 7 Wayne State University School of Medicine, Detroit, MI 48202 USA

**Keywords:** Biochemistry, Biotechnology, Computational biology and bioinformatics, Neuroscience, Biomarkers, Diseases, Medical research

## Abstract

Traumatic brain injury (TBI) is a major cause of mortality and disability worldwide, particularly among individuals under the age of 45. It is a complex, and heterogeneous disease with a multifaceted pathophysiology that remains to be elucidated. Metabolomics has the potential to identify metabolic pathways and unique biochemical profiles associated with TBI. Herein, we employed a longitudinal metabolomics approach to study TBI in a weight drop mouse model to reveal metabolic changes associated with TBI pathogenesis, severity, and secondary injury. Using proton nuclear magnetic resonance (^1^H NMR) spectroscopy, we biochemically profiled post-mortem brain from mice that suffered mild TBI (N = 25; 13 male and 12 female), severe TBI (N = 24; 11 male and 13 female) and sham controls (N = 16; 11 male and 5 female) at baseline, day 1 and day 7 following the injury. ^1^H NMR-based metabolomics, in combination with bioinformatic analyses, highlights a few significant metabolites associated with TBI severity and perturbed metabolism related to the injury. We report that the concentrations of *taurine*, *creatinine*, *adenine*, *dimethylamine*, *histidine*, *N-Acetyl aspartate*, and *glucose 1-phosphate* are all associated with TBI severity. Longitudinal metabolic observation of brain tissue revealed that mild TBI and severe TBI lead distinct metabolic profile changes. A multi-class model was able to classify the severity of injury as well as time after TBI with estimated 86% accuracy. Further, we identified a high degree of correlation between respective hemisphere metabolic profiles (r > 0.84, p < 0.05, Pearson correlation). This study highlights the metabolic changes associated with underlying TBI severity and secondary injury. While comprehensive, future studies should investigate whether: (a) the biochemical pathways highlighted here are recapitulated in the brain of TBI sufferers and (b) if the panel of biomarkers are also as effective in less invasively harvested biomatrices, for objective and rapid identification of TBI severity and prognosis.

## Introduction

Traumatic brain injury (TBI), a frequently encountered neurological condition in pediatric, neurologic, emergency room, military, and sports medicine practices, is the cause of trauma from an external mechanical force on the brain resulting in Glasgow Coma Scale of 3 to 15^1–6^. It is a major cause of mortality and disability worldwide, affecting all ages^7^. TBI constitutes a major health and socioeconomic burden throughout the world^8^. TBI has the highest incidence and prevalence of all neurological diseases affecting more than 50 million people worldwide each year and causing a global economic burden of approximately $400 billion annually^9^. According to the 2019 Global Disease Burden study stroke resulted in 6.5 million deaths and 143 million disability-adjusted life years in 2019^10^. The Centers for Disease Control and Prevention (CDC) estimates that 1.7 million people in the United States suffer from TBI annually and one third of survivors experience long-term disability^11^. Older adults have the greatest number and rates of TBI-related hospitalizations and deaths. According to the CDC, people 75 or older account for nearly one-third of TBI-related hospitalizations and more than one-quarter of all TBI-related deaths. Other populations also struggle with traumatic brain injury in greater proportions, according to CDC data. Men are three times as likely to die from a TBI than women at a rate of 28.3 per 100,000. Over 450,000 US service members were diagnosed with a traumatic brain injury between 2000 and 2021—approximately 80% of which occurred in non-deployed settings^12^. Moreover, the total economic burden associated with TBI is estimated to be $76.5 billion annually, with approximately 75% of cases being athletes and military personnel^13,14^.

There is currently a lack of knowledge to completely elucidate the complex and dynamic pathophysiology of TBI which hinders progress towards the development of novel strategies for TBI diagnosis and treatment. Therefore, it is essential to decipher the underlying pathological mechanisms of TBI, particularly during the acute phase, so that effective treatments can be developed to reduce morbidity and mortality. In that respect, the alterations in energy metabolism^15^ within 24 h after TBI point to the role they play in the development of the delayed, secondary post-traumatic tissue damage. Regional time and site-specific alterations in ATP, glucose, and lactate content were found in the cortical and subcortical structures, in a rat model of closed head injury (CHI). Moreover, there have been extensive efforts studying the molecular mechanism underlying TBI to better understand disease pathophysiology and to develop intervention and therapeutic strategies^16–20^. In the clinical laboratory setting, the diagnostic measures for TBI include brain edema, alterations in intracranial pressure (ICP) and cerebral blood flow, and metabolic changes^21,22^. Of these changes, investigation of metabolic alterations in the biofluid or point of injury is pivotal for the clinical diagnosis. Measurement and characterization of the metabolites at the cellular level as a consequence of metabolic activity have great potential to both sensitively and specifically identify the severity and phenotype of TBI, track pathophysiology and metabolic state, and ultimately aid in the development of therapeutic treatment plans^23,24^. Current noninvasive metabolic biomarkers (either metabolite, peptide, or protein based) for TBI include S100 calcium binding protein B (S100B)^25–28^, glial fibrillary acidic protein (GFAP)^29^, neuron-specific enolase (NSE)^29,30^, sphingolipid (SPL)^31^, ubiquitin carboxy-terminal hydrolase-L1^32^, plasma DNA^33^, myelin-based protein^34^, and medium-chain fatty acids^35^.

As one of the two major analytical platforms used in metabolomics, NMR spectroscopy has made significant advances in basic research, method development, and applications across a wide range of disciplines^36^. Many unique features of NMR, including excellent reproducibility, high quantitative nature, the ability to unambiguously identify unknown metabolites, and the ability to identify metabolites using intact biological samples, compensate for the relatively low sensitivity^37^. Additionally, NMR monitors the flow of labeled substrates such as ^2^H, ^13^C, and ^15^N from the nucleus to downstream metabolites, providing a unique opportunity to reliably identify active metabolic pathways and measure metabolic fluxes and activities enzymatic. Therefore, NMR-based metabolomic applications have taken advantage of the unique advantages of NMR, which greatly complements mass spectrometry^38^. NMR-based metabolomics has great potential for the comprehensive screening of hundreds of metabolites and is a powerful technique that can provide a global picture of many metabolic processes underlying complex and multifactorial diseases such as neurodegenerative diseases^39–44^.

In the current study we aim to apply a ^1^H NMR-based metabolomics approach to explore brain metabolic changes in a Closed Head Injury (CHI) weight drop mouse model of TBI with varying severity and at different time points so that we can systematically analyzed the dynamic changes of the brain metabolome. Through analysis of longitudinal samples, it would be possible to find out prognostic markers are directly related to disease progression and that their levels should be restored to baseline upon disease recovery. In this model, injury severity was assessed by the neurobehavioral (NSS) test, as well as by T2-weighted MRI abnormalities, motor and cognitive function^45^. It demonstrated that assessment of the neurobehavioral parameter, NSS at 1 h is a reliable tool for evaluation of injury severity as it correlates with the later post-injury motor dysfunction, cognitive damage, and brain-damage characteristics as depicted by T2-weighted MRI. The combined assessment of neurobehavioral and cognitive function along with MRI is most useful in evaluating severity of injury and potential for recovery after TBI. We hypothesize that TBI will lead to a cascade of metabolic effects in the brain that will eventually be detectable in less invasive biofluids such as blood and urine which can be used in diagnosis, scoring severity and prognosis of TBI.

## Experimental procedures

### Animal models

#### Animals

The study was performed according to the Institutional Animal Care and Use Committee guidelines in compliance with National Institutes of Health (NIH; Bethesda, MD) guidelines and were validated by the Joseph Sagol Neuroscience Center’s Institutional Animal Care and Use committee. We confirm that all the experiments and procedures performed complied with ARRIVE guidelines^46^. Adult C57bl male and female mice (Harlan, Jerusalem, Israel) ages 8 to 10 weeks and weighing 23–27 g were used. The animals were housed in 5 mice/cage during the testing period under controlled temperature and light conditions and had access to food and water ad libitum. Mice were maintained at a constant temperature (22 ± 2 °C) and a 12-h light/dark cycle.

#### Closed-head injury model

**Figure 1:**
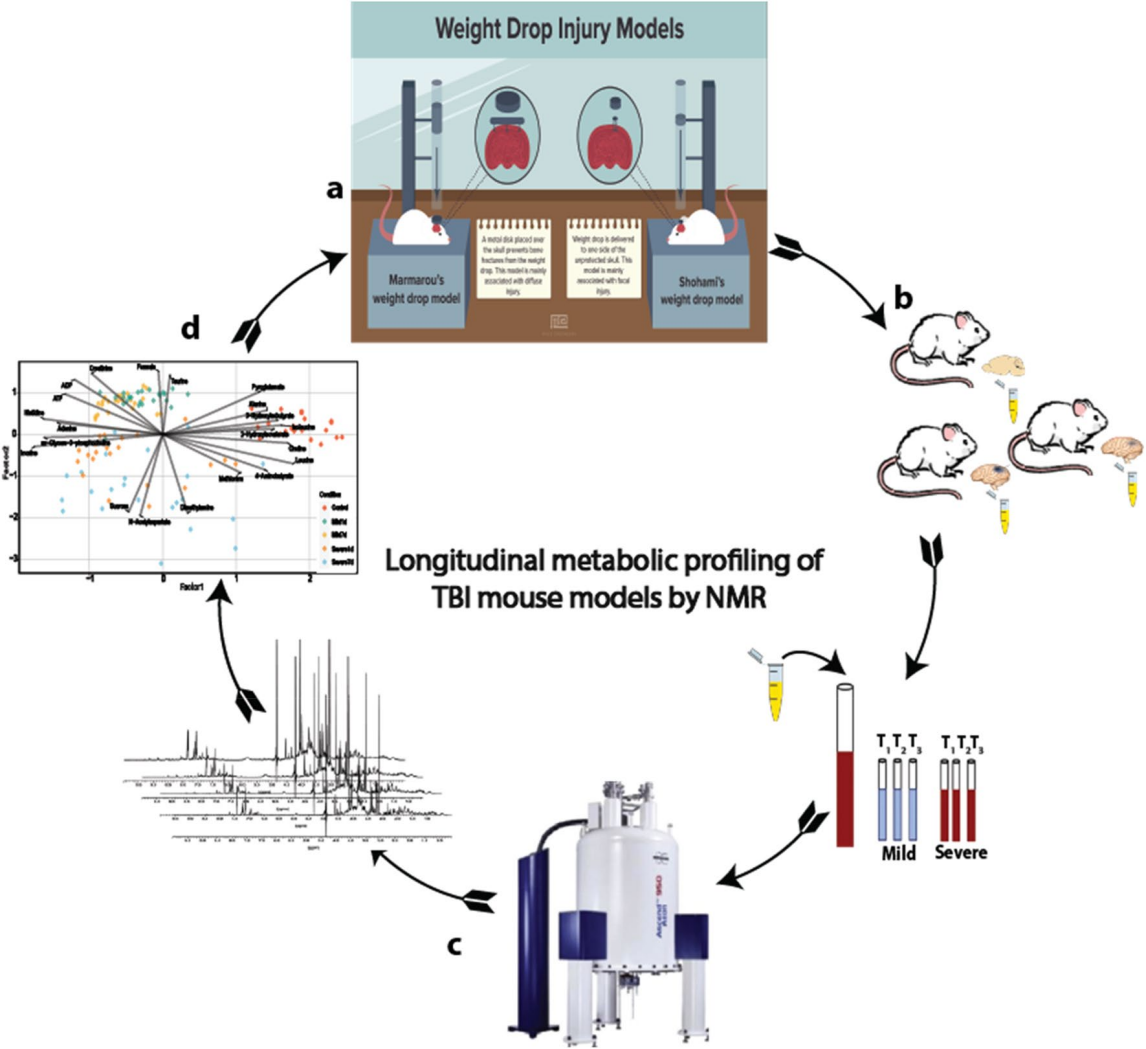
Workflow implemented to explore brain metabolic alteration due to TBI. (**a**) Closed head injury model, experimental CHI was induced by using a weight-drop device method developed^110^ and modified^111^ by Shohami et al. (**b**) Sample collection, metabolite extraction and sample preparation for ^1^H NMR based metabolomics; (**c**) data collection and characterization of brain metabolic signatures by ^1^H NMR spectroscopy; (**d**) longitudinal statistical modelling and bioinformatic analysis of ^1^H NMR metabolomics data.

Experimental closed-head injury (CHI) was induced by using a weight-drop device previously developed in Dr. Shohami’s laboratory^47^ (Fig. 1A). Under 3% isoflurane anesthesia and supplementary oxygen (confirmed by the loss of response to pinch of paw), a midline longitudinal incision was performed, and the skull was exposed, and the retracted skin was manually fixed at the surface under the falling rod after identification of the correct landmarks and localization of the area to be impacted. The left anterior frontal area was identified, a tipped Teflon cone (2 mm diameter) was placed 2 mm lateral to the midline and 2 mm caudal to the left coronal suture, and a metal rod weighing 95 g was dropped down on the cone from a pre-adjusted height. The height of the free-fall weight was determined by the weight of the mouse and the desired CHI severity, as defined by the Neurological Severity Score (NSS). This is a 10-point scale that assesses the functional neurological status of mice based on the presence of reflexes and the ability to perform motor and behavioral tasks such as beam walking, beam balance, and spontaneous locomotion^48^. One point is awarded for failing to perform a task; thus, a normal, uninjured mouse score. Thus, the left hemisphere is directly affected by the weight drop injury and the right hemisphere is non-lesioned. Immediately after trauma, the mice received supporting oxygenation with 95% O_2_ for no longer than 2 min and were then returned to their cages. Controls (namely, sham operated animals) were anesthetized, their scalps were incised, but they were not subjected to CHI, and were sacrificed at the same time points as the CHI experimental groups, namely at 24 h or 7 days post injury. It is noteworthy that < 5% of the injured mice were excluded from the study, mostly because of death by apnea within minutes of injury.

### ^1^H NMR spectroscopy

#### Sample preparation

Sample preparation was carried out via blinding fashion. Frozen tissue samples (~ 0.25 g) were lyophilized and milled to a fine powder under liquid nitrogen to limit heat production and were stored at − 80 °C prior to preparation. For ^1^H NMR 50 mg samples were extracted in 50% methanol/water (1 g/mL) in a sterile 2 mL Eppendorf tube. The samples were mixed for 20 min and sonicated for 20 min, and the protein was removed by centrifugation at 13,000×*g* at 4 °C for 30 min. Supernatants were collected, dried under vacuum using a Savant DNA Speedvac (Thermo Scientific, USA) and reconstituted in 285 μL of 50 mM potassium phosphate buffer (pH 7.0), 30 μL of Sodium 2,2-dimethyl-2-silapentane-5-sulfonate (DSS) and 35 μL of D_2_O. 200 μL of the reconstituted sample was transferred to a 3 mm Bruker NMR tube for analysis. All samples were housed at 4 °C in a thermostatically controlled SampleJet autosampler (Bruker-Biospin, USA) and heated to room temperature over 3 min prior to analysis by NMR^49^ (Fig. 1B).

#### Data collection and metabolic profiling by ^1^H NMR spectroscopy

During the course of the study, all the experimental protocols were approved by the Beaumont Institutional Review Board (IRB# 2015-281). All 1D ^1^H NMR data were recorded at 300 (± 0.5) K on a Bruker ASCEND HD spectrometer operating at 600 MHz (Bruker-Biospin, USA) proton frequency coupled with a 5 mm TCI cryoprobe in randomized order (Fig. 1C). For each sample, 256 transients were collected as 64 k data points with a spectral width of 12 kHz (20 ppm), using a pulse sequence (Bruker pulse program: pusenoesypr1d) developed by Mercier et al.^50^ and inter-pulse delay of 9.65 s. During the data collection 180-s temperature equilibration period, fast 3D shimming using the z-axis profile of the ^2^H NMR solvent signal, receiver gain adjustment was applied. The free induction decay signal was zero filled to 128 k and exponentially multiplied with a 0.1 Hz line broadening factor. All the one-dimensional NMR spectra were phase-corrected manually, while the baseline correction was performed automatically with a fifth order polynomial fitting routine. DSS was used as internal reference standard (0 ppm) for spectra calibration and for precise quantitation. All spectra were processed and analyzed using Chenomx NMR Suite (ver. 8.3, Edmonton, Canada).

### Statistical analysis

All analyses were performed using R programming language (R version 4.1.3; 2022-03-10) unless indicated otherwise (Fig. 1D).

#### Data processing

Several quality control steps were performed prior to normalization. Any 0-concentration values in the raw dataset were replaced with NA. The concentration values were log transformed. Any metabolite containing more than 30% missing values was removed from further analysis. The remaining missing values were imputed using the K nearest neighbors algorithm (k = 5). Technical outlier samples were identified using principal component analysis of the scaled and centered value matrix. Samples deviating more than three SDs away from the mean (at 99.7% confidence) of any of the first 3 principal components were considered outliers and removed from further analysis. Finally, the data was normalized sample-wise using the quantile normalization method.

#### Differential abundance analysis using linear models

Linear regression modeling was performed using brain site, gender, and various conditions (trauma severity, and time after trauma) as covariates (Fig. 1D). Using remove unwanted variation (RUV) package possible additional unknown covariates were modelled^51^. A linear model without intercept was fitted to each metabolite using the limma package in R^52^. Mouse ID was added to the models as a blocking factor to model variance within the samples originating from the same mouse. Empirical Bayes correction was then applied to model fits. The Student’s t-test as implemented in each pair-wise comparisons. The contrasts. Fit() function from limma package was used to compare severe trauma to control and mild trauma to control group at each time point. For comparison, resulting p values were adjusted with Benjamini–Hochberg correction for multiple testing and those with FDR q < 0.05 were deemed significant. In order to construct the metabolite heatmap across all conditions (severe, mild, control; 24 h, 7 days), only the metabolites that were found to be significantly differentially abundant in any of the conditions were taken into account. Metabolite abundance measures were centered to 0 mean and scaled to unit standard deviation. Mean metabolite abundance was computed for each metabolite in each of the conditions. R package pheatmap^53^ was used to draw the heatmap and cluster the metabolites using hierarchical clustering. Potential differences in metabolic profiles between left (lesioned) and right (non-lesioned) hemispheres were investigated. Further, overall Pearson correlation between metabolic profile changes in the right and left side of the brain was calculated.

#### Metabolite set enrichment analysis

Metabolites were ranked using the rankFeatures method from R package multiGSEA^54^. Metabolites without known Human metabolome database (HMDB) ID were removed from the ranked list. The Kyoto Encyclopedia of Genes and Genomes (KEGG)^55^ pathway database for metabolomic data was used to evaluate pathway enrichment using R package fGSEA^56^. Enrichment scores of each pathway were calculated using fgseaMultilevel() function with 10,000 permutations. R package pathview^57^ was used to visualize the top 5 significant pathways in each contrast. The sign of Normalized Enrichment Score (NES) was used to determine if a certain metabolic pathway is up-regulated or down-regulated and significance of the perturbation is assessed by nominal p-values.

#### Construction of multi-class predictors

Prior to multi-class modeling, the unnormalized log transformed and imputed metabolic profiles of all samples were clustered using hierarchical clustering. Four samples for which clustering dendrogram height was above 0.15 (corresponding to Pearson r = 0.75) were considered outliers and were removed from further modeling. Next, the data were quantile normalized and clustered again. Additional three outliers were identified and removed from further analysis. R package caret^58^ was used to train and evaluate the predictive models. Five random data partitions were created, each with 80% of the data used for model training and 20% used for model validation. Within each training set metabolites with zero and near-zero variation were removed. We trained a random forest predictor on the training data subset using 3 times repeated tenfold cross-validation. The best model for each partition was selected using logLoss metric. The final classifier of each training set was validated on the corresponding validation set. The predictions were then pooled together to get an overall unbiased accuracy of the predictor. The final classifier model was built on all the available data using random forest with recursive feature elimination^59^.

## Results

### Animals

There was a total of 65 mice of which 25 had mild TBI, 24 had severe TBI and 16 were sham controls (Table S1). Half of each TBI group was sacrificed at 24 h and half at 7 days post TBI to determine if the effects of TBI were measurable as a result of trauma and secondary injury. The left and right side of each mouse brain was harvested separately to determine if there were any differences in their respective metabolomes resultant from the lesioned and non-lesioned hemispheres. There was no significant difference in body weight of mild TBI, severe TBI and sham controls; mean (SD) = 25.1 (1.93) g versus 24.7 (1.90) g, versus 26. 3 (1.34) (chi-squared, p = 0.745). Similarly, no significant differences in body weight were observed when early TBI mice (24 h sacrifice) compared to the late group (sacrificed after 7 days). Weight mean (SD) were 26.5 (1.25) g versus 23.8 (1.46) g, (t-test, p = 0.13, respectively).

### Statistical analysis of metabolomics data

**Figure 2:**
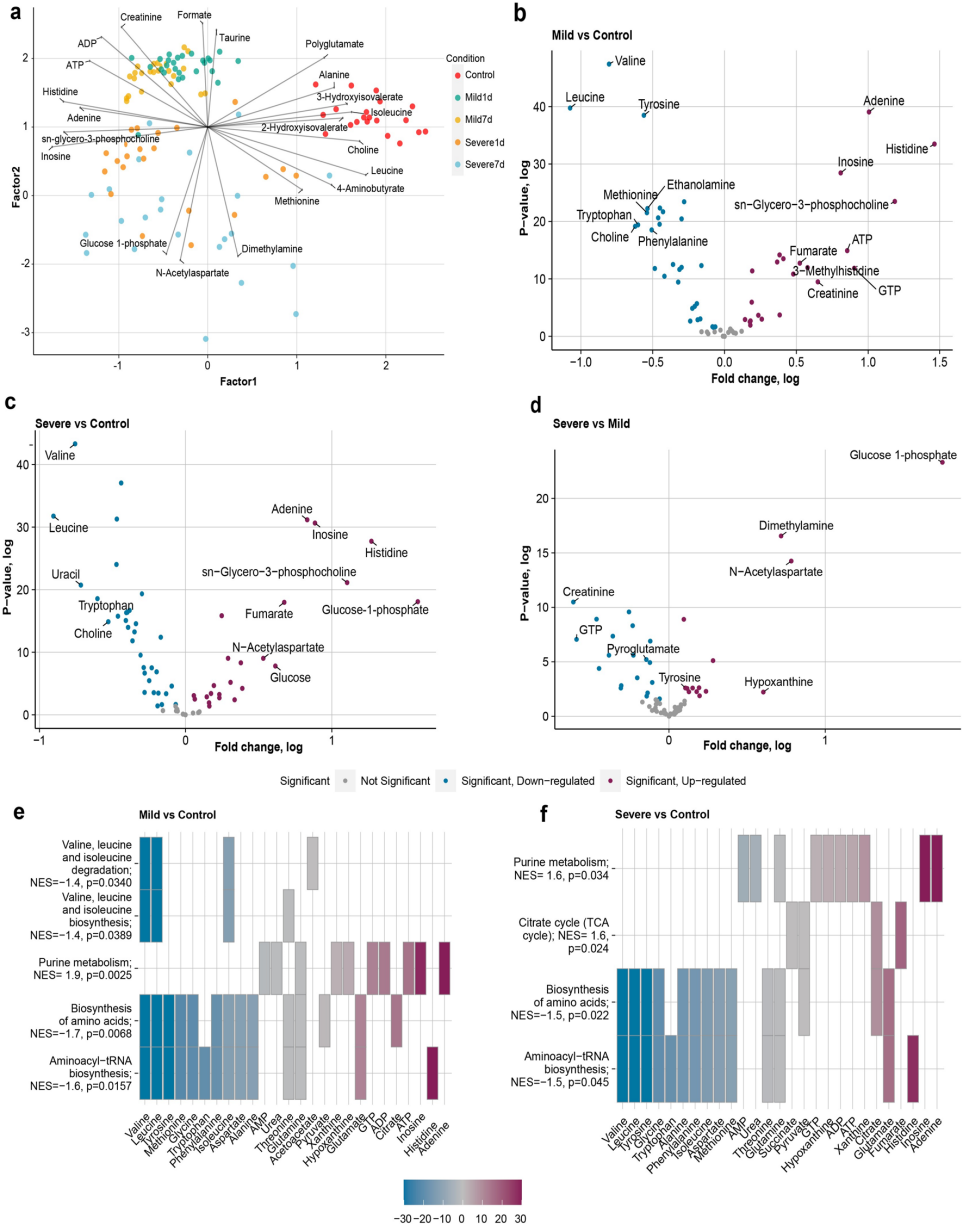
(**a**) Score plot provided by factor analysis based on ^1^H NMR based metabolomics data associated with TBI biomarkers for disease severity and recovery exhibits an excellent separation. Factor analysis reveals the most discriminative metabolites driving the separation mainly are inosine, adenine, 2-hydroxyisovelarate, choline, isoleucine, and sn-glycero-3-phosphocholine. (**b**) Volcano plot comparing the metabolite concentration in mild TBI brain vs. sham control brain with fold change and associated statistical significance revealed *Valine, Leucine, Tyrosine, Adenine and Histidine* were the most affected metabolites; (**c**) Volcano plot comparing the metabolite concentration in severe TBI brain vs. sham control brain with fold change and associated statistical significance revealed *Valine, Leucine, Inosine, Uracil and Adenine* were the most affected metabolites; (**d**) Volcano plot comparing metabolite concentration in severe TBI brain vs. mild TBI brain has shown that *Glucose-1-phosphate, Dimethylamine, NAA, and Creatinine* were the metabolites showing statistically significantly largest concentration change; (**e**) enrichment analysis showing significantly perturbed metabolic pathways when mild TBI group is compared to sham controls and (**f**) enrichment analysis showing significantly perturbed metabolic pathways when the severe TBI group is compared to sham controls.

Using 1D ^1^H NMR a total of 66 metabolites were accurately identified and quantified in PM brain tissue. Using Principal Component Analysis (PCA) we first sought to determine if there was any systematic variation or outliers in our dataset and three samples from severe TBI group were found to be outlier (Fig. S1). Following outlier detection we first performed the analyses separately for each brain side and found a strong correlation of the results were attained (Table S2). Therefore, we pooled metabolomics data from each hemisphere together and included a blocking factor into subsequent linear models. To better understand the variation structure in the dataset, factor analysis (FA) was also conducted. Factor analysis highlighted factor 1 (26.07%) and factor 2 (9.87%) as being the most informative, explaining the maximum amount of variation between the sample groups and a distinct separation between control, mild TBI, and severe TBI cases on the score plots along the first two factors (Fig. 2A). Factor analysis indicates the most discriminative metabolites driving the separation are *inosine*, *adenine*, *2-hydroxyisovelarate, choline*, *isoleucine*, and *sn-glycero-3-phosphocholine*. Similarly, component analysis revealed that high concentrations of *formate*, *taurine*, *creatinine*, *ADP* and low concentrations of *dimethylamine*, *N-acetyl aspartate*, and *glucose-1-phosphate* leading to tight clustering of the mild TBI group (24 h and 7 days) while clearly separating them from the rest of the samples. For severe TBI cases (24 h and 7 days) high concentrations of methionine, *dimethylamine*, *glucose-1-phosphate*, and lower concentrations of *pyroglutamate*, *ADP*, *alanine*, *creatinine* was responsible for the observed separation (Fig. 2A). Heatmap analysis of the data indicated that over the recovery course and according to the level of trauma, metabolites are differentially abundant (Fig. S2).

To identify metabolites showing significant concentration changes, we fitted least squares linear regression model that accounted for possible unknown source of unwanted variation and potential cofounders such as sex and brain site. Linear models comparing sham controls with mild TBI cases (both 24 h and 7 days) revealed that 49 of the 66 metabolites show statistically significant concentration changes (FDR < 0.05) of which 21 were increased and 28 at decreased concentrations (Table S3). For this comparison, the most significantly affected metabolites were found to be *valine*, *leucine*, *tyrosine*, *inosine*, *histidine*, and *sn-Glycero-3-phosphocholine* (Fig. 2B). When the same analysis was run separately on each hemisphere, 5 and 3 of the significantly changed metabolites were found to be specific to the left hemisphere and the right hemisphere, respectively (Fig. S3A,B). Table S4 lists the results of the linear model comparing metabolic profile differences between the initial injury (24 h) and post-injury (7 days) of mild TBI cases. The results indicate that, 7 metabolites were found to have significantly (FDR < 0.05) altered concentrations and the most significantly affected metabolites include *creatine,* 3*-hydroxybutyrate, choline*, *ATP*, *fumarate,* and *glucose* (Fig. S4). The linear models comparing the mean concentrations of all metabolites between sham control and severe TBI cases indicate 50 metabolites with significant concentration differences (FDR < 0.05) (Table S5). In this comparison, the six most influential metabolites were found to be *valine*, *inosine, fumarate, pyroglutamate, tyrosine*, and *leucine* (Fig. 2C). Further analysis of this comparison revealed that, 6 and 8 of the significantly affected metabolites were specific to the left hemisphere and the right hemisphere, respectively (Fig. S3C,D). When comparing the initial injury of severe TBI (24 h) with post-injury of severe TBI (7 days) groups, 12 metabolites reached statistically significant differences (FDR < 0.05) (Table S6). Of those metabolites, the top six metabolites were found to be *dimethylamine*, *3-methylhistidine*, *N-acetyl-L-aspartic acid (NAA), valine*, *hypoxanthine*, and *glucose* (Fig. S5). The final linear model holistically compared (regardless of initial or post injury) metabolic profile changes associated with mild and severe TBI. In this comparison, a total of 33 metabolites showed significant concentration changes between the groups (Table S7). *Glucose-1-phosphate*, *dimethylamine*, *NAA*, *pyroglutamic acid*, *carnitine*, and *creatinine* were identified by linear model to be the most affected metabolites (FDR < 0.05) between mild TBI and severe TBI cases (Fig. 2D, Fig. S6). Comparison between the hemispheres showed that of the significantly affected metabolites 12 and 9 were specific to left hemisphere and the right hemisphere, respectively (Fig. S3E,F).

### Metabolite set enrichment analysis

Metabolite set enrichment analysis (MSEA) revealed that multiple biochemical pathways were perturbed in mild TBI cases (both 24 h and 7 days) compared to sham controls. These include *purine metabolism (NES* = 2.0,* p* = 0.0003)*, valine, leucine and isoleucine biosynthesis (NES* =  − 1.5,* p* = 0.01111), *valine, leucine and isoleucine degradation* (*NES* =  − 1.5,* p* = 0.0093), *aminoacyl-tRNA biosynthesis,* and *biosynthesis of amino acids* (*NES* =  − 1.5,* p* = 0.0035) were nominally perturbed (Fig. 2E). Interestingly, a subsequent MSEA investigating metabolic pathways comparing the initial injury of mild TBI (24 h) with post-injury of mild TBI (7 days) revealed that only *pyruvate metabolism* (*NES* = 1.4525,* p* = 0.0361) and *arginine and proline metabolism* (*NES* = 1.4404,* p* = 0.0421) were the most affected (Table S8). In this instance, although not statistically significant, *glycerophospholipid metabolism*, *inflammatory mediator regulation of TRP channels* were found to be influenced as well.

MSEA of the mean metabolite concentration data of severe TBI and control cases identified *purine metabolism* (*NES = 1.6, p-nom* = 0.0231) and *citrate metabolism (TCA cycle)* (*NES* = 1.5, *p-nom* = 0.0342) to be significantly upregulated, whereas *alanine, leucine and isoleucine degradation* (NES =  − 1.6, p-nom = 0.0056), *valine, leucine and isoleucine biosynthesis* (NES =  − 1.5, p-nom = 0.0087) and *biosynthesis of amino acids* (NES =  − 1.5, p-nom = 0.0371) were significantly downregulated between control and severe TBI cases (Fig. 2F). MSEA comparing the initial injury of severe TBI (24 h) with post-injury of severe TBI (7 days) identified four metabolic pathways to be profoundly perturbed but only one of them reached nominal statistical significance (Table S9). These include *glycolysis/gluconeogenesis* (*NES* =  − 1. 5945,* p* = 0.0075), *valine, leucine, and isoleucine degradation* (*NES* = 1.4001, *p-nom* = 0.0619), *arginine and proline metabolism* (*NES* = 1.3661, *p-nom* = 0.0861) and *primary bile acid biosynthesis* (*NES* =  − 1.345, *p-nom* = 0.1037).

The final MSEA comparing all mild TBI (both initial and post-injury) and all severe TBI cases (both initial and post-injury) highlights *glycerophospholipid (NES* = 1.4798, *p-nom* = 0.0021), *taste transduction* (*NES* = 1.4285, *p-nom* = 0.039) and *primary bile-acid metabolism* (*NES* = -1.2285, *p-nom* = 0.0184) to be significantly perturbed (Table S10).

### Longitudinal modeling

**Figure 3:**
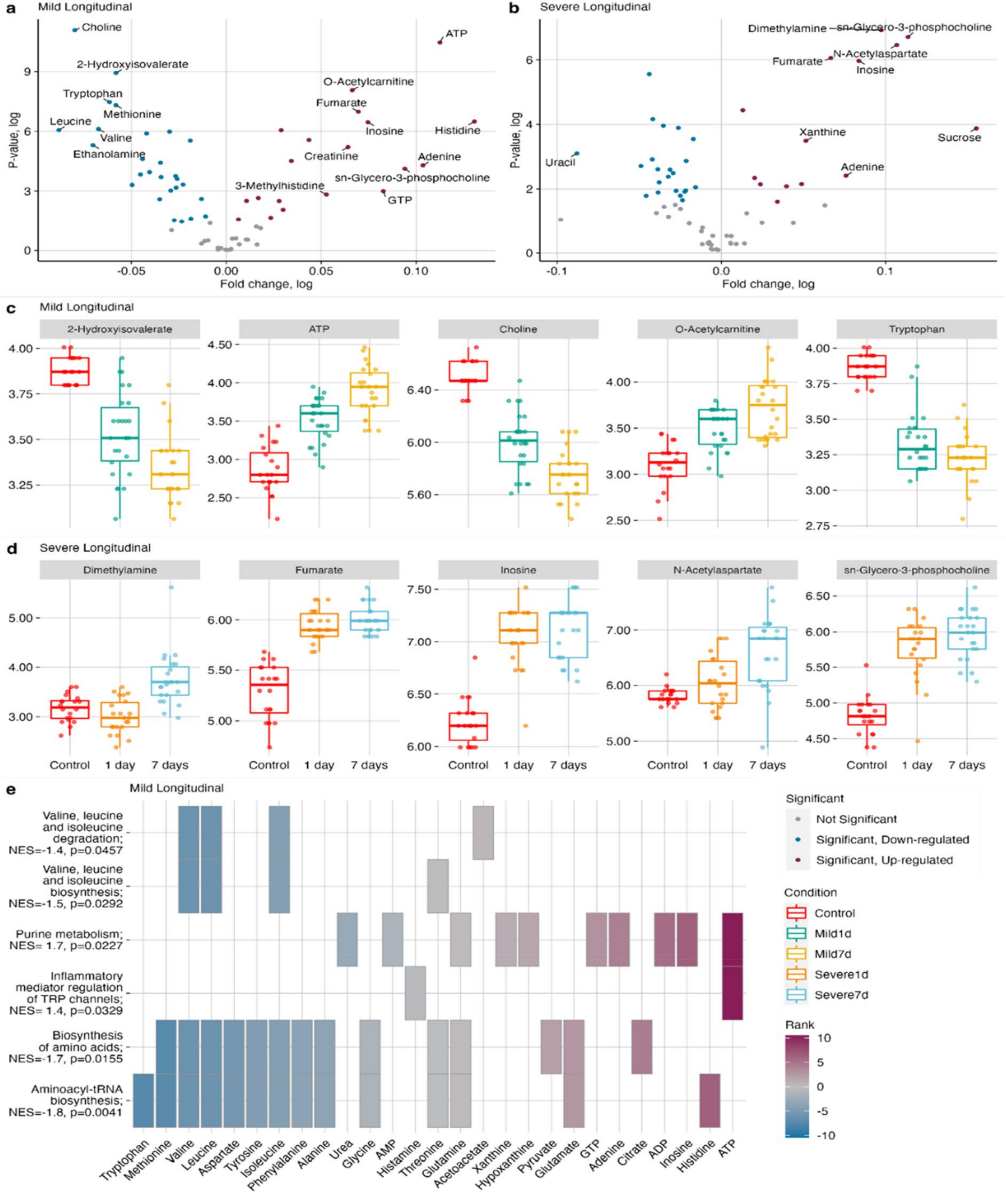
(**a**) Volcano plot comparing longitudinal metabolic profile change in mild TBI brain vs. sham control brain, metabolites displayed at the top-left and top-right corner are the metabolites showing the largest concentration change with the greatest statistical significance; the colors indicated metabolites are not significant, significantly down regulated or significantly up-regulated; (**b**) Volcano plot comparing longitudinal metabolic profile change in severe TBI vs. sham control brain, metabolites displayed at the top-left and top-right corner are the one metabolites showing the largest concentration change with the greatest statistical significance, the colors indicated metabolites are not significant, significantly down regulated or significantly up-regulated, (**c**) the trajectory of top five metabolites driving the separation in longitudinal model utilizing mild TBI brain metabolomics data; condition for each time point color coded, (**d**) the trajectory of top five metabolites provided by longitudinal modelling utilizing severe TBI brain metabolomics data, condition for each time point color coded, and (**e**) enrichment analysis based on longitudinal metabolic profile change showing significantly perturbed metabolic pathways in the case of mild TBI group compared to sham controls.

Volcano plots of the longitudinal models highlight 46 and 36 metabolites to be at significantly different concentrations between mild TBI and severe TBI with their baselines, respectively (Fig. 3A,B). In mild TBI the top five most longitudinally effected metabolites were *2-hydroxyisovalarete*, *ATP*, *choline*, *O-acetylcarnitine*, and *tryptophan*. Similarly, for the longitudinal observation in severe TBI the top five most perturbed metabolites include *inosine*, *fumarate*, *dimethylamine*, *sn-Glycero-3 phosphocholine*, and *N-acetyl aspartate (NAA).* It is noteworthy that metabolites follow entirely different trends between mild and severe TBI over the course of secondary injury (Fig. 3C,D). In parallel to cross-sectional comparison the longitudinal investigation of metabolomics data revealed that in mild TBI the perturbed metabolic pathways include the upregulation of *purine metabolism (NES* = 1.7, *p-nom* = 0.0227), *inflammatory mediator of TRP channel metabolism (NES* = 1.4,* p-nom* = 0.0329), and down regulation *of valine, leucine and isoleucine biosynthesis (NES* =  − 1.5, *p-nom* = 0.0292), *valine, leucine and isoleucine degradation (NES* =  − 1.4, *p-nom* = 0.0457), *Aminoacyl t-RNA biosynthesis (NES* =  − 1.8, *p-nom* = 0.0041) and *biosynthesis of amino acids (NES* =  − 1.7, *p-nom* = 0.0155) (Fig. 3E). In the case of MSEA based on longitudinal metabolic profiles of severe TBI, the two most significant pathways are Aminoacyl *t-RNA biosynthesis* (NES =  − 1.5, p-nom = 0.0445) and ether lipid metabolism (NES = 1.3, p-nom = 0.0577) (Table S11). In mild TBI case, when longitudinal analysis was run separately on each hemisphere, 9 and 8 of the significantly changed metabolites were found to be specific to the left hemisphere and the right hemisphere, respectively. Similarly, in severe TBI case the corresponding number was found to be 4 and 37, respectively (Figs. S7A, S8A). Investigation of the longitudinal metabolic profile changes in each hemisphere has yielded striking results. Firstly, following TBI, metabolites have followed the same trend in both hemispheres. Secondly, the concentrations of metabolites at the site of injury (left hemisphere) were higher than those of the uninjured hemisphere without exception (Figs. S7B, S8B).

### Multi-class modeling

**Figure 4:**
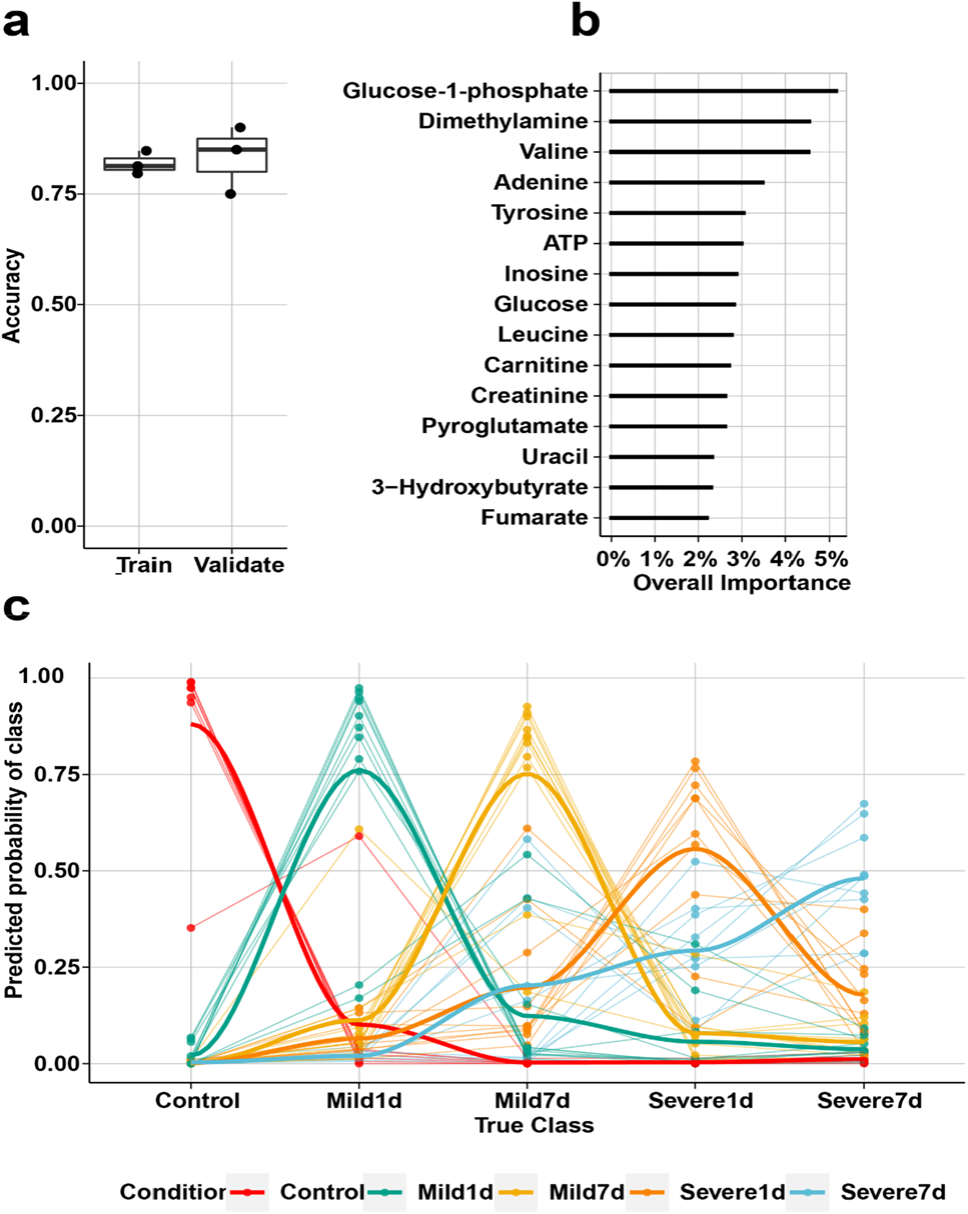
(**a**) Multi-class models based on all brain metabolomics data; Predictor accuracy for both training set (based on 80% of the whole metabolomics data), and validation set (based on the 20% of the whole metabolomics data), (**b**) top 15 most important metabolites explaining majority of the variation and driving the separation in multi-class model, (**c**) predictive performance of multi-class model; true class of each sample was compared with predicted probability of each class. The color code indicates each group.

We performed hierarchical clustering analysis and identified seven outlier samples, which were removed from further analysis (Fig. S9A). After their removal, we observed that the samples were randomly distributed across the clusters for both gender and brain hemispheres, however, severity and time after trauma were separated from each other (Fig. S9B). It is clearly indicated that there was not an effect of sex on the metabolic profile. Next, we built a predictive model using the random forest algorithm. The multi-class model provided average predictive accuracies of 81% and 85% in training and validation datasets, respectively (Fig. 4a). The predictive performance of the model was further assessed by evaluating the class prediction probabilities for each sample group and time point (Fig. 4c), which revealed that the model is able to distinguish the groups and timepoints with high accuracy. The final classifier was trained using the Recursive Feature Elimination (RFE) algorithm on the complete dataset. In this instance, the highest estimated accuracy of 88.5% was achieved when 68 features were utilized. The top 15 most important metabolites are shown in Fig. 4b.

## Discussion

In the current study, we evaluated ^1^H NMR-based metabolic signatures of brain tissue in a closed head injury, weight drop mouse model of TBI at an early (24 h) and late (7 days) phases following injury. To the best of our knowledge, this is the first reported mouse model study applying ^1^H NMR to biochemically profile brain tissue with the aim of monitoring longitudinal metabolic changes to improve our understanding of the neurobiological pathophysiology of TBI. Although our understanding still remains incomplete, it has been reported that following TBI some degree of functional and structural reorganization may occur in both the ipsilesional and contralesional hemispheres during recovery to regain motor functionality and cognition^60,61^. One should be aware that in a closed head injury, it is likely that both hemispheres would receive some level of damage from the primary mechanical insult as the force gets distributed across the skull despite being locally applied to one side. Therefore, in an attempt to better understand the secondary injury process, we collected tissue samples from both hemispheres. It is our hope that investigation of the difference in metabolite alterations in ipsilesional and contralesional hemispheres, and their modulations during appropriate time windows in mild and severe TBI, will facilitate the understanding of the disease and help identification of reliable biomarkers and the development of novel therapeutic strategies for the treatment of TBI.

Based on linear models and enrichment analysis comparing sham controls with mild TBI cases (both 24 h and 7 days), we detected a substantial alteration in the concentration of several metabolic pathways (p < 0.05). We highlighted significant disturbances in *purine metabolism*, *valine, leucine and isoleucine biosynthesis*, *valine, leucine and isoleucine degradation*, *aminoacyl-tRNA biosynthesis*, and *biosynthesis of amino acids*.

Of the significantly altered metabolic pathways, *purine metabolism* was markedly perturbed during TBI recovery. Previous studies have reported on the neuroprotective role purines have in the CNS^62,63^. Purines such as hypoxanthine are reaction intermediates in adenosine metabolism and in the formation of nucleic acids via the salvage pathway. It is formed from the breakdown of inosine which we have shown to have neuroprotective and immunomodulatory effects in the same CHI model used in the present study^64^. Herein we also report higher levels of inosine in the mice that have suffered a TBI, which suggest it may be suppressing macrophage, neutrophil, and lymphocyte activity and attenuating levels of pro-inflammatory mediators during recovery^65^.

Energy impairment^15^ is considered a secondary metabolism of TBI^66^ and involves numerous purine nucleotides such as AMP, GMP and NAD all of which are required for RNA and DNA synthesis. We hypothesize that perturbations in purine metabolism may have additional consequences such as those directly related to energy crisis and energy failure, which is the outcome of multiple mechanisms, including classical ischemia^67^, diffusion hypoxia^68^, mitochondrial dysfunction^69^, increased energy needs from excitotoxicity, seizure activity, and spreading depolarization^70^.

Disruption to both *valine*, *leucine and isoleucine biosynthesis* and *degradation* highlight the roles of branched chain amino acids (BCAA) in TBI pathology. Consistent with our findings, Jeter et al.^71^ previously reported that circulating levels of valine, isoleucine, leucine, and other key metabolites in blood were significantly reduced in patients with mild TBI compared to healthy sham controls. This effect was even more profound in the blood of patients with severe TBI. In the brain, BCAAs serve as important metabolic precursors that are essential for the synthesis of proteins and neurotransmitters, such as dopamine, serotonin, and norepinephrine^72^. An important source of nitrogen, BCAAs also promote the synthesis of essential brain metabolites, namely glutamate and glutamine^73^. In addition, BCAAs have been demonstrated to play a critical role in recovery from TBI in rodents^74^. It has been reported that BCAA supplementation can restore net synaptic efficiency and hippocampal function, resulting in improved cognitive performance.

Aminoacyl-tRNAs are produced by aminoacyl-tRNA synthetases, and expression of the protein associated with the pro-inflammatory mediator macromolecular tRNA synthetase complex (MSCp43) has been shown to be a biomarker in brain injury to distinguish between traumatic and ischemic brain injuries^75,76^. Therefore, the upregulation of Aminoacyl-tRNAs synthesis observed herein may be related to the control of inflammation following brain injury. Interestingly, an enrichment analysis comparing severe TBI group with healthy controls showed that the metabolic pathways associated with *purine metabolism*, *Aminoacyl-tRNA biosynthesis*, *the biosynthesis of amino acids* and *the TCA cycle* are particularly impaired. Of these metabolic pathways, only the TCA cycle has been found to be specific to severe TBI; the other pathways are also linked to mild TBI.

Changes in cerebral metabolism following TBI have overwhelmingly been ascribed to either disruptions of glycolysis or end point mitochondrial function^15,77^. It is generally accepted that changes in mitochondrial function are critical components of the secondary injury cascade initiated by TBI that promotes neurodegeneration and limits neuroregeneration^78^. However, less is known about the initiation and progression of the TCA cycle and how it may contribute to mitochondrial function and, ultimately, cerebral metabolism as a result of TBI. Indeed, the fate of TCA cycle whether it is up regulated^13,79^ or down regulated^80,81^ following the brain injury is quite controversial. Significant upregulation of the TCA cycle may be an attempt to preserve energy metabolism, and it may have upstream beneficial consequences on several energy-requiring pathways, some of which are critical for cell recovery after TBI.

Finally, to better understand how perturbations in neurometabolism are associated with TBI severity we undertook a pair-wise enrichment analysis between mild and severe TBI cases. Strikingly, in this pair-wise comparison, glycerophospholipid metabolism and bile-acid metabolism were found to be significantly perturbed (p < 0.05). Previous studies in rodent serum^23,82^, brain imaging^83,84^, and human plasma studies^85,86^ have shown that the concentration of individual glycerophospholipids varies with severity and time of injury. Glycerophospholipids are a major component of neural cell membranes and have been shown to be altered in several neurological disorders including TBI due to the disruption of those membranes^87^. In the acute stage of TBI, excessive release of glutamate initiates an excitotoxic cascade, and the resulting buildup of intracellular Ca^2+^ activates phospholipases which degrade glycerophospholipids into diacylglycerols (DAGs) which are important second messenger signaling lipids^88^. Moreover, downstream actions attributed to glycerophospholipid-generated second messengers include inflammation, membrane transport, cell differentiation and proliferation, and apoptotic processes. Therefore, one can speculate that significant changes in glycerophospholipids may indicate injury-induced inflammation, increased need for membrane repair, or alternatively, less cell signaling and/or turnover^81,89^. Moreover, the endocannabinoids (eCBs) such as 2-arachidonoyl-glycerol, are produced from phospholipid precursors when intracellular Ca2+ is elevated, and as we have shown to be neuroprotective after TBI, using the same model of CHI as in the present study^90–92^. Therefore, one may also speculate that changes in their levels after TBI may be part of the brain’s self-defense mechanism, which aims to reduce the extent of the damage and facilitate motor and cognitive recovery.

Traumatic brain injury disrupts the “brain-gut-microbiome axis”, a well-balanced network formed by the brain, gastrointestinal tract, and gut microbiome, which has a complex effect: brain damage alters the composition of the microbiome; altered microbiome influences TBI severity, neuroplasticity, and metabolic pathways through various bacterial metabolites^93–96^. In support of our findings where we report a disturbance in Bile acid metabolism, You et al. demonstrated that TBI can induce alterations to the gut microbiota, and showed that bacterial diversity experienced a time-dependent change after TBI which eventually led to significant changes in the bile acid profile^97^. The decrease in the concentration of primary and secondary bile acids in both feces and plasma were associated with intestinal inflammation.

### Longitudinal modeling

In addition to the previously described cross-sectional comparisons, we also sought to understand the longitudinal phenotypical changes associated with of mild and severe TBI brains at subacute time points corresponding to ongoing secondary cell degradation as well as the time course of post-traumatic inflammatory and oxidative stress cascades. Observation of such dynamic changes has potential to provide deeper insights into the secondary injury of the brain associated with mild and severe TBI and enable better prognosis and the development of future treatment modalities. Interestingly, after the initial injury, the observed metabolite pattern was quite different for mild and severe TBI, respectively. Our longitudinal metabolic observation of brain tissue revealed that mild TBI and severe TBI lead distinct metabolic profile change.

We hypothesize that multiple metabolic cascades are disrupted as a consequence of the initial injury, then the brain tries to maintain a resting level of specific metabolites but because of significantly advancing hallmark pathology it cannot reach initial values as observed in healthy, cognitively intact individuals.

Our longitudinal modeling highlighted *inflammatory mediator regulation of transient receptor potential (TRP) channel* and *ether lipid metabolism* as the two major pathways that are associated with mild and severe TBI neuronal recovery, respectively. Numerous studies underline the critical role of the post-injury inflammatory response in long-term recovery from TBI^98^. As such, TRP channels play an important role in the regulation of inflammation through sensory function and the release of neuropeptides. Recent studies have shown the involvement of TRP channels in processes associated with TBI induced oxidative stress, inflammation, calcium and zinc penetration, and cell death. Using the same CHI as in the present study, we have shown that TRPC1-sensitive mechanisms are involved in TBI pathology, and that inhibition of this channel by carvacrol enhances recovery and should be considered for further studies in animal models and humans^99^. Therefore, we speculate that downregulation of TRP prevents neuronal death by reducing zinc and calcium influx during the recovery period following TBI.

TBI disrupts tissue architecture and leads to complex changes in all tissue components, especially lipids, as the brain is predominantly made up of various lipids species. Ojo et al. recently reported a significant increase in total phosphatidylethanolamine ester (ePE) and phosphatidylcholine ester (ePC) in the cortex and hippocampus in a post-traumatic model of TBI^100^. These lipids, particularly the plasmalogens, play a vital role in vesicle formation and membrane fusion and may also affect neurotransmitter release following TBI^101^. Therefore, excessive production of these types of lipids can have devastating effects on neuronal signaling and can lead to dysfunction in neurotransmitter systems ubiquitous throughout the brain, such as the glutamatergic systems. Glutamate excitotoxicity has been previously reported in in vivo TBI models and changes in etherPE, among other factors, may contribute to this biochemical event after injury^102^. Notably, the non-lesioned right hemisphere has been potentially damaged due to secondary causes, including neuroinflammation and BBB disruption. These secondary causes of damage likely affect the left hemisphere which received the primary lesion. Surprisingly, both hemispheres show similar longitudinal metabolic changes, which warrant further study.

### Multi-class models

Although the predictive value of the Glasgow Coma Scale (GCS) has been well-studied, there are currently no biomarkers known to predict patient outcomes in routine clinical use^103^. Moreover, it is reported that 50% of concussions in the USA, approximately 3.8 million cases go unreported, underscoring the enormous challenge associated with accurate TBI diagnosis^104^. Therefore, development of reliable, objective, minimally invasive biomarkers for TBI will have tremendous influence in the practice of civilian and military medicine^105^. Despite the fact that in the past few decades protein-based biomarkers have been the center of attention^106^ for diagnosis of TBI, these biomarkers generally suffer from lack of sensitivity and specificity. This is due to a combination of inherent (genetic, etc.) and technical (collection and processing) variability, and the inability to pass an intact blood brain barrier (BBB)^66^ Metabolites on the other hand are more readily able to pass an intact BBB and are thus much more independent from fluctuating and immeasurable confounders related to BBB dysfunction, which is one of the hallmarks of TBI pathophysiology. The multi-class model presented here, using the top fifteen most discriminative features as selected by RFE, holds great promise with high predictive accuracy (88.5%) of classification of all groups at once enabling a real-world approach. This observation demonstrates that the TBI profiles are separated with a high degree of sensitivity, and that the model provides a robust group prediction.

If these reported neuronal metabolites changes are observable in more accessible biomatrices such as blood or urine, then there is a strong potential for developing both prognostic and diagnostic biomarkers of TBI. This would allow earlier intervention and potential sequential patient monitoring throughout their recovery. Further, it would allow the treating clinicians to make an objective diagnosis and determine if said patient needs additional monitoring or is capable of returning to their normal activities^107,108^.

A general limitation of our study includes the modest number of animals used; however, even with such a sample number we were able to develop models with excellent predictive accuracies and reveal specifics about the post-TBI recovery period in terms of the dynamic metabolic changes. Following the power calculation of the available metabolomics data, with 25 mild TBI, 24 severe TBI and 16 sham control mice, this study was well-powered to detect most, if not all, “huge” and “very large” effects after false discovery rate multiple testing adjustments. We have even been able to detect the majority of true large and medium effects as well, indicating current data have been able to provide the power originally intended for (over 90% power; a fold change (FC) of 1.2 with a 30% coefficient of variation; a confidence interval width of ± 0.05 for the AUC assuming the true AUC is 0.90)^109^. Further, of the two main analytical platforms (NMR and MS), only an NMR-based metabolomics approach has been utilized here which has its own advantages and limitations. Therefore, to provide a more in-depth view of the metabolic changes associated with TBI, future studies should aim to use two or more complimentary techniques simultaneously to provide a more in depth, holistic oversight of TBI metabolism.

## Conclusions

In conclusion, using an NMR-based metabolomics approach and for the first time, we report on the longitudinal metabolic changes in the brain of a TBI mouse model and link these changes to severity and recovery times. Further, a cross-sectional metabolic profile comparison between sham control vs. mild TBI, sham control vs. severe TBI and severe vs. mild TBI demonstrates the potential of metabolomics for identifying potential biomarkers of TBI while giving a previously unreported insight into the underlying pathogenesis of the disorder such as impact of severity on the gut-brain axes. As this was a proof-of-concept study, the small sample sizes preclude evaluation of temporal changes. While this study primarily focused on using brain extracts to identify biomarkers of TBI it is important to note that many of these metabolites can be noninvasively detected and quantified in the peripheral tissues such as serum and urine. This suggests that these findings and at least some of the biomarkers identified herein may be recapitulated in peripheral tissues leading to non-invasive, robust clinical test for TBI. These novel findings may also lay the groundwork for the development of effective therapeutic strategies and improvement in clinical TBI management.

### Ethics approval

The study was performed according to the Institutional Animal Care and Use Committee guidelines in compliance with National Institutes of Health (NIH; Bethesda, MD) guidelines.

### Supplementary Information

Supplementary Information.

## Data Availability

The metabolomics and metadata reported in this paper are available at MetaboLights Archive (https://www.ebi.ac.uk/metabolights/mysubmissions?status=PRIVATE) via the MetaboLights partner repository with the data set no. MTBSXXX Username: ali.yilmaz@beaumont.org and study ID is MTBLS.
