# Supplementary material for: The longitudinal biochemical profiling of TBI in a drop weight model of TBI
Source: Sci Rep. 2023 Dec 14;13:22260. doi: 10.1038/s41598-023-48539-x (PMC10721861; doi:10.1038/s41598-023-48539-x)
Supplement: Supplementary file 1 — Supplementary Information. [file 41598_2023_48539_MOESM1_ESM.docx]

**The longitudinal biochemical profiling of TBI in a drop weight model of TBI.**

Ali Yilmaz, PhD^1,2^, Sigal Liraz-Zaltsman ^3-5^**,** Esther Shohami^4^, Juozas Gordevičius^6^, Ieva Kerševičiūtė^6^, Eric Sherman^7^, Ray O. Bahado-Singh^2^, and Stewart Francis Graham, PhD^1,2*^

1- Metabolomics Department, Beaumont Research Institute, Beaumont Health, Royal Oak, MI 48073, USA.

2- William Beaumont School of Medicine, Oakland University, Rochester, MI 48073, USA

3-Department of Pharmacology, the Institute for Drug Research, The Hebrew University of Jerusalem, Jerusalem, Israel.

4-The Joseph Sagol Neuroscience Center, Sheba Medical Center, Ramat-Gan, Israel.

5-Institute for Health and Medical Professions, Department of Sports Therapy, Ono Academic College, Qiryat Ono, Israel.

6- VUGENE LLC, Grand Rapids, MI, United States.

7- Wayne State University School of Medicine, Detroit, Michigan, USA

*****Correspondence: Stewart Francis Graham; Email: stewart.graham@beaumont.edu

; Tel: +1-248-712-4595

Academic Editor: name

Received: date; Accepted: date; Published: date

**Supplementary Table 1:** Design of the experiment indicating how many animals have been used to access the impact of brain site, sex, time points and disease severity on metabolic profile change associated with traumatic brain injury (TBI).

| **Samle number** | **Brain Region** | **TimePoint** | **Severity** | **Gender** |
| --- | --- | --- | --- | --- |
| 1 | Left hemisphere | 7d | Severe | Female |
| 2 | Left hemisphere | 7d | Severe | Female |
| 3 | Left hemisphere | 7d | Severe | Female |
| 4 | Left hemisphere | 7d | Severe | Female |
| 5 | Left hemisphere | 7d | Severe | Female |
| 6 | Left hemisphere | 7d | Severe | Female |
| 7 | Left hemisphere | 7d | Severe | Female |
| 8 | Right hemisphere | 7d | Severe | Female |
| 9 | Right hemisphere | 7d | Severe | Female |
| 10 | Right hemisphere | 7d | Severe | Female |
| 11 | Right hemisphere | 7d | Severe | Female |
| 12 | Right hemisphere | 7d | Severe | Female |
| 13 | Right hemisphere | 7d | Severe | Female |
| 14 | Right hemisphere | 7d | Severe | Female |
| 15 | Left hemisphere | 7d | Severe | Male |
| 16 | Left hemisphere | 7d | Severe | Male |
| 17 | Left hemisphere | 7d | Severe | Male |
| 18 | Left hemisphere | 7d | Severe | Male |
| 19 | Left hemisphere | 7d | Severe | Male |
| 20 | Left hemisphere | 7d | Severe | Male |
| 21 | Right hemisphere | 7d | Severe | Male |
| 22 | Right hemisphere | 7d | Severe | Male |
| 23 | Right hemisphere | 7d | Severe | Male |
| 24 | Right hemisphere | 7d | Severe | Male |
| 25 | Right hemisphere | 7d | Severe | Male |
| 26 | Right hemisphere | 7d | Severe | Male |
| 27 | Left hemisphere | 1d | Severe | Female |
| 28 | Left hemisphere | 1d | Severe | Female |
| 29 | Left hemisphere | 1d | Severe | Female |
| 30 | Left hemisphere | 1d | Severe | Female |
| 31 | Left hemisphere | 1d | Severe | Female |
| 32 | Left hemisphere | 1d | Severe | Female |
| 33 | Right hemisphere | 1d | Severe | Female |
| 34 | Right hemisphere | 1d | Severe | Female |
| 35 | Right hemisphere | 1d | Severe | Female |
| 36 | Right hemisphere | 1d | Severe | Female |
| 37 | Right hemisphere | 1d | Severe | Female |
| 38 | Right hemisphere | 1d | Severe | Female |
| 39 | Left hemisphere | 1d | Severe | Male |
| 40 | Left hemisphere | 1d | Severe | Male |
| 41 | Left hemisphere | 1d | Severe | Male |
| 42 | Left hemisphere | 1d | Severe | Male |
| 43 | Left hemisphere | 1d | Severe | Male |
| 44 | Right hemisphere | 1d | Severe | Male |
| 45 | Right hemisphere | 1d | Severe | Male |
| 46 | Right hemisphere | 1d | Severe | Male |
| 47 | Right hemisphere | 1d | Severe | Male |
| 48 | Right hemisphere | 1d | Severe | Male |
| 49 | Left hemisphere | 7d | Mild | Female |
| 50 | Left hemisphere | 7d | Mild | Female |
| 51 | Left hemisphere | 7d | Mild | Female |
| 52 | Left hemisphere | 7d | Mild | Female |
| 53 | Left hemisphere | 7d | Mild | Female |
| 54 | Left hemisphere | 7d | Mild | Female |
| 55 | Right hemisphere | 7d | Mild | Female |
| 56 | Right hemisphere | 7d | Mild | Female |
| 57 | Right hemisphere | 7d | Mild | Female |
| 58 | Right hemisphere | 7d | Mild | Female |
| 59 | Right hemisphere | 7d | Mild | Female |
| 60 | Right hemisphere | 7d | Mild | Female |
| 61 | Left hemisphere | 7d | Mild | Male |
| 62 | Left hemisphere | 7d | Mild | Male |
| 63 | Left hemisphere | 7d | Mild | Male |
| 64 | Left hemisphere | 7d | Mild | Male |
| 65 | Left hemisphere | 7d | Mild | Male |
| 66 | Left hemisphere | 7d | Mild | Male |
| 67 | Right hemisphere | 7d | Mild | Male |
| 68 | Right hemisphere | 7d | Mild | Male |
| 69 | Right hemisphere | 7d | Mild | Male |
| 70 | Right hemisphere | 7d | Mild | Male |
| 71 | Right hemisphere | 7d | Mild | Male |
| 72 | Right hemisphere | 7d | Mild | Male |
| 73 | Left hemisphere | 1d | Mild | Female |
| 74 | Left hemisphere | 1d | Mild | Female |
| 75 | Left hemisphere | 1d | Mild | Female |
| 76 | Left hemisphere | 1d | Mild | Female |
| 77 | Left hemisphere | 1d | Mild | Female |
| 78 | Left hemisphere | 1d | Mild | Female |
| 79 | Left hemisphere | 1d | Mild | Female |
| 80 | Right hemisphere | 1d | Mild | Female |
| 81 | Right hemisphere | 1d | Mild | Female |
| 82 | Right hemisphere | 1d | Mild | Female |
| 83 | Right hemisphere | 1d | Mild | Female |
| 84 | Right hemisphere | 1d | Mild | Female |
| 85 | Right hemisphere | 1d | Mild | Female |
| 86 | Right hemisphere | 1d | Mild | Male |
| 87 | Left hemisphere | 1d | Mild | Male |
| 88 | Left hemisphere | 1d | Mild | Male |
| 89 | Left hemisphere | 1d | Mild | Male |
| 90 | Left hemisphere | 1d | Mild | Male |
| 91 | Left hemisphere | 1d | Mild | Male |
| 92 | Left hemisphere | 1d | Mild | Male |
| 93 | Right hemisphere | 1d | Mild | Male |
| 94 | Right hemisphere | 1d | Mild | Male |
| 95 | Right hemisphere | 1d | Mild | Male |
| 96 | Right hemisphere | 1d | Mild | Male |
| 97 | Right hemisphere | 1d | Mild | Male |
| 98 | Right hemisphere | 1d | Mild | Male |
| 99 | Right hemisphere | None | Control | Male |
| 100 | Right hemisphere | None | Control | Male |
| 101 | Right hemisphere | None | Control | Male |
| 102 | Right hemisphere | None | Control | Male |
| 103 | Right hemisphere | None | Control | Male |
| 104 | Left hemisphere | None | Control | Male |
| 105 | Left hemisphere | None | Control | Male |
| 106 | Left hemisphere | None | Control | Male |
| 107 | Left hemisphere | None | Control | Male |
| 108 | Left hemisphere | None | Control | Male |
| 109 | Right hemisphere | None | Control | Female |
| 110 | Right hemisphere | None | Control | Female |
| 111 | Right hemisphere | None | Control | Female |
| 112 | Right hemisphere | None | Control | Female |
| 113 | Right hemisphere | None | Control | Female |
| 114 | Left hemisphere | None | Control | Female |
| 115 | Left hemisphere | None | Control | Female |
| 116 | Left hemisphere | None | Control | Female |
| 117 | Left hemisphere | None | Control | Female |
| 118 | Left hemisphere | None | Control | Female |

**Supplementary Table 2:** The calculated the correlation of fold change between the left and right hemispheres and corresponding significance for each pairwise comparison.

| **Comparison** | **Pearson correlation** | **statistic** | **p.values** | **Lower confidence intervall** | **Higher confidence intervall** |
| --- | --- | --- | --- | --- | --- |
| **Mild TBI 1d vs. Control** | 0.9257 | 19.88 | 2e-16 | 0.88 | 0.95 |
| **Mild TBI 7d vs. Control** | 0.9568 | 26.73 | 2e-16 | 0.93 | 0.97 |
| **Severe TBI 1d vs. Control** | 0.7267 | 8.60 | 2e-16 | 0.59 | 0.82 |
| **Severe TBI 7d vs. Control** | 0.8728 | 14.53 | 2e-16 | 0.80 | 0.92 |
| **Mild TBI (all) vs. Control** | 0.9576 | 27.02 | 2e-16 | 0.93 | 0.97 |
| **Severe TBI (all) vs. Control** | 0.8448 | 12.83 | 2e-16 | 0.76 | 0.90 |

**Supplementary Table 3:** The results of linear model comparing metabolic profile differences between all Mild TBI (24 hours and 7days) with controls.

| **Metabolites** | **logFC** | **AveExpr** | **P.Value** | **adj.P.Val** | **B** |
| --- | --- | --- | --- | --- | --- |
| Valine | -0.8086152 | 5.01563969 | 1.05E-31 | 6.94E-30 | 61.52503 |
| Leucine | -1.0767622 | 4.85165998 | 2.38E-26 | 7.84E-25 | 49.19875 |
| Tyrosine | -0.5641349 | 4.39424205 | 5.43E-24 | 1.19E-22 | 43.77049 |
| Inosine | 0.79830781 | 6.91219303 | 2.58E-23 | 4.25E-22 | 42.21503 |
| Histidine | 1.42073622 | 5.53765732 | 6.11E-20 | 8.07E-19 | 34.45414 |
| Sn-Glycero-3-phosphocholine | 1.15315041 | 5.70635968 | 5.03E-19 | 4.8E-18 | 32.35018 |
| Adenine | 1.17178312 | 7.79664914 | 5.09E-19 | 4.8E-18 | 32.3396 |
| Choline | -0.6194173 | 6.02149574 | 1.98E-17 | 1.64E-16 | 28.68618 |
| ATP | 0.87533906 | 3.37403277 | 6.78E-17 | 4.97E-16 | 27.46196 |
| ADP | 0.43231814 | 3.3774816 | 1.26E-15 | 8.34E-15 | 24.54949 |
| 3-Hydroxyisobutyrate | -0.4154287 | 2.90047377 | 1.99E-15 | 1.19E-14 | 24.09692 |
| Anserine | -0.4884409 | 4.95469821 | 5E-14 | 2.75E-13 | 20.89074 |
| Fumarate | 0.51774877 | 5.7854433 | 2.9E-13 | 1.47E-12 | 19.1462 |
| 2-Hydroxyisovalerate | -0.4490694 | 3.52794025 | 2.59E-12 | 1.15E-11 | 16.97218 |
| Isoleucine | -0.3651277 | 4.23208092 | 2.62E-12 | 1.15E-11 | 16.96194 |
| 4-Aminobutyrate | -0.4391623 | 7.87200503 | 8.9E-12 | 3.67E-11 | 15.74961 |
| Phenylalanine | -0.5286144 | 4.28833214 | 1.08E-11 | 4.18E-11 | 15.56093 |
| Alanine | -0.304711 | 6.72841021 | 1.17E-11 | 4.28E-11 | 15.48153 |
| Tryptophan | -0.5950459 | 3.39692074 | 2.17E-11 | 7.52E-11 | 14.86917 |
| Citrate | 0.35942931 | 4.7019504 | 2.56E-11 | 8.43E-11 | 14.70507 |
| GTP | 0.95368552 | 2.68055059 | 4.87E-11 | 1.53E-10 | 14.06735 |
| Ethanolamine | -0.5710188 | 5.72278698 | 8.76E-11 | 2.63E-10 | 13.48626 |
| Pyroglutamate | -0.2670074 | 7.23067125 | 3.11E-10 | 8.93E-10 | 12.23433 |
| Acetone | -0.3184374 | 2.80797213 | 7.5E-10 | 2.06E-09 | 11.36603 |
| Glutamate | 0.22218106 | 8.7156094 | 9.85E-10 | 2.6E-09 | 11.09776 |
| Creatinine | 0.67437319 | 5.77285604 | 3.57E-09 | 9.07E-09 | 9.827131 |
| Dimethylamine | -0.5144738 | 3.04276947 | 1.03E-07 | 2.52E-07 | 6.524972 |
| IMP | 0.31248588 | 2.80582284 | 4.09E-07 | 9.65E-07 | 5.178582 |
| Methionine | -0.5239695 | 4.19976733 | 5.77E-07 | 1.31E-06 | 4.843265 |
| O-Acetylcarnitine | 0.48730068 | 3.47098433 | 3.22E-06 | 7.09E-06 | 3.170382 |
| O-Phosphocholine | -0.2145827 | 6.43169893 | 6.01E-06 | 1.28E-05 | 2.566212 |
| Glycerol | -0.2300268 | 6.94766745 | 8.17E-06 | 1.68E-05 | 2.268716 |
| Isobutyrate | -0.2840891 | 2.4103972 | 1.13E-05 | 2.25E-05 | 1.958661 |
| Glycine | -0.3470949 | 7.56197292 | 1.27E-05 | 2.47E-05 | 1.839758 |
| Aspartate | -0.1576725 | 8.52058269 | 1.37E-05 | 2.58E-05 | 1.769262 |
| Carnitine | 0.20939475 | 4.12140061 | 1.68E-05 | 3.09E-05 | 1.570176 |
| 3-Methylhistidine | 0.51659489 | 4.55753333 | 3.07E-05 | 5.48E-05 | 0.991444 |
| Glucose | 0.37007348 | 4.38835611 | 0.000643 | 0.001116 | -1.90462 |
| Propylene.glycol | -0.3445123 | 2.57487709 | 0.000678 | 0.001147 | -1.95468 |
| Acetate | -0.1088716 | 8.83960908 | 0.001357 | 0.002239 | -2.60539 |
| Uracil | -0.4123387 | 5.51728167 | 0.002361 | 0.0038 | -3.12055 |
| Dimethyl.sulfone | -0.1630075 | 2.16167864 | 0.006349 | 0.009977 | -4.02986 |
| Succinate | 0.17759263 | 5.38374682 | 0.01094 | 0.016792 | -4.52241 |
| Lactate | 0.03829818 | 9.29903302 | 0.013861 | 0.020792 | -4.73459 |
| UDP.glucose | 0.23800052 | 3.67977419 | 0.015386 | 0.022567 | -4.82774 |
| Xanthine | 0.18768972 | 3.93287281 | 0.018743 | 0.026892 | -5.00298 |
| Histamine | -0.1772707 | 3.12562888 | 0.021075 | 0.029595 | -5.10662 |
| 3-Hydroxybutyrate | 0.22549653 | 4.24557677 | 0.022981 | 0.031599 | -5.18284 |
| N-Acetylaspartate | -0.2430703 | 5.91718872 | 0.028352 | 0.038189 | -5.36674 |
| AMP | -0.0742355 | 3.38118513 | 0.060276 | 0.079564 | -6.01285 |
| Creatine | -0.0303972 | 8.77998274 | 0.09819 | 0.127069 | -6.4152 |
| Serine | 0.05477142 | 6.76889521 | 0.104991 | 0.133258 | -6.46918 |
| Pyruvate | 0.12840595 | 4.93076454 | 0.157364 | 0.195962 | -6.78733 |
| myo.Inositol | 0.04704925 | 8.4173641 | 0.164609 | 0.201188 | -6.82175 |
| Pantothenate | -0.0589829 | 4.0791588 | 0.221451 | 0.262127 | -7.04262 |
| Niacinamide | 0.07731328 | 5.83851477 | 0.222411 | 0.262127 | -7.04575 |
| Methanol | 0.02751703 | 7.97244661 | 0.249644 | 0.289061 | -7.12847 |
| Urea | -0.0810272 | 5.7140526 | 0.264157 | 0.300592 | -7.16818 |
| Taurine | 0.02812858 | 8.81147868 | 0.294634 | 0.329591 | -7.24334 |
| Formate | 0.07724938 | 4.59045768 | 0.31952 | 0.34845 | -7.2977 |
| Glutamine | -0.1266753 | 6.75459739 | 0.322052 | 0.34845 | -7.30292 |
| Threonine | -0.0954125 | 6.07922969 | 0.355538 | 0.374079 | -7.36719 |
| Hypoxanthine | 0.25255692 | 3.42422593 | 0.357075 | 0.374079 | -7.36995 |
| UDP-galactose | 0.02095058 | 3.29541878 | 0.691162 | 0.705725 | -7.71931 |
| Glucose-1-Phosphate | -0.0667713 | 2.91962535 | 0.695032 | 0.705725 | -7.72141 |
| Acetoacetate | 0.00955378 | 3.89231318 | 0.904117 | 0.904117 | -7.79185 |

**Supplementary Table 4:** The results of the linear model comparing metabolic profile differences between the initial injury of Mild TBI (24 hours) and post-injury (7days) of mild TBI.

| **Metabolites** | **logFC** | **AveExpr** | **P.Value** | **adj.P.Val** | **B** |
| --- | --- | --- | --- | --- | --- |
| Creatine | 0.080516 | 8.779983 | 7.04E-05 | 0.002582 | 1.379795 |
| 3-Hydroxybutyrate | -0.42901 | 4.245577 | 7.82E-05 | 0.002582 | 1.28148 |
| Choline | -0.25108 | 6.021496 | 0.000199 | 0.004384 | 0.415926 |
| ATP | 0.349506 | 3.374033 | 0.000333 | 0.005499 | -0.05783 |
| Fumarate | 0.241327 | 5.785443 | 0.000438 | 0.005784 | -0.30916 |
| Glucose | -0.39846 | 4.388356 | 0.000588 | 0.006465 | -0.57821 |
| 2-Hydroxyisovalerate | -0.18509 | 3.52794 | 0.002943 | 0.027748 | -2.03949 |
| Urea | -0.20038 | 5.714053 | 0.010698 | 0.08826 | -3.18482 |
| Ethanolamine | -0.20247 | 5.722787 | 0.018808 | 0.137928 | -3.67488 |
| O-Acetylcarnitine | 0.235044 | 3.470984 | 0.028746 | 0.189724 | -4.03752 |
| Carnitine | 0.098854 | 4.121401 | 0.049126 | 0.294759 | -4.48654 |
| Pyruvate | 0.176181 | 4.930765 | 0.070295 | 0.386622 | -4.77958 |
| N-Acetylaspartate | 0.199423 | 5.917189 | 0.090891 | 0.431538 | -4.98532 |
| myo-Inositol | 0.060952 | 8.417364 | 0.092732 | 0.431538 | -5.00119 |
| Pyroglutamate | -0.06805 | 7.230671 | 0.101203 | 0.431538 | -5.07008 |
| Glutamine | 0.222717 | 6.754597 | 0.104615 | 0.431538 | -5.09607 |
| Dimethyl.sulfone | 0.100185 | 2.161679 | 0.112365 | 0.43624 | -5.1518 |
| Aspartate | -0.05376 | 8.520583 | 0.14895 | 0.499083 | -5.36746 |
| UDP-glucose | -0.14845 | 3.679774 | 0.153592 | 0.499083 | -5.39051 |
| Pantothenate | -0.07311 | 4.079159 | 0.156646 | 0.499083 | -5.40524 |
| Acetone | 0.071523 | 2.807972 | 0.158799 | 0.499083 | -5.41543 |
| ADP | 0.065835 | 3.377482 | 0.184993 | 0.55498 | -5.52808 |
| Methionine | -0.1344 | 4.199767 | 0.204852 | 0.567244 | -5.60181 |
| 3-Hydroxyisobutyrate | -0.05989 | 2.900474 | 0.213596 | 0.567244 | -5.63166 |
| Tryptophan | -0.10622 | 3.396921 | 0.214865 | 0.567244 | -5.63587 |
| Acetoacetate | 0.100295 | 3.892313 | 0.23818 | 0.60461 | -5.70832 |
| Glycerol | -0.05839 | 6.947667 | 0.268317 | 0.639138 | -5.79015 |
| Methanol | -0.02758 | 7.972447 | 0.280116 | 0.639138 | -5.81913 |
| Uracil | -0.1525 | 5.517282 | 0.283576 | 0.639138 | -5.82734 |
| Inosine | 0.071061 | 6.912193 | 0.290517 | 0.639138 | -5.84343 |
| UDP-galactose | -0.05709 | 3.295419 | 0.312003 | 0.664265 | -5.89028 |
| Taurine | -0.02538 | 8.811479 | 0.375837 | 0.771302 | -6.00756 |
| O-Phosphocholine | -0.04196 | 6.431699 | 0.385651 | 0.771302 | -6.02317 |
| Dimethylamine | 0.08004 | 3.042769 | 0.408316 | 0.792614 | -6.05714 |
| Citrate | 0.040958 | 4.70195 | 0.429586 | 0.801062 | -6.08659 |
| Histidine | 0.105261 | 5.537657 | 0.436943 | 0.801062 | -6.09628 |
| Histamine | 0.056882 | 3.125629 | 0.483695 | 0.862807 | -6.15237 |
| Isoleucine | -0.0334 | 4.232081 | 0.501125 | 0.870374 | -6.17109 |
| 3-Methylhistidine | 0.079909 | 4.557533 | 0.53015 | 0.892341 | -6.1999 |
| Creatinine | 0.068102 | 5.772856 | 0.544947 | 0.892341 | -6.21354 |
| Tyrosine | 0.027389 | 4.394242 | 0.554333 | 0.892341 | -6.22184 |
| AMP | -0.02189 | 3.381185 | 0.601422 | 0.945091 | -6.25968 |
| Formate | 0.038174 | 4.590458 | 0.644712 | 0.978177 | -6.28937 |
| Acetate | -0.01546 | 8.839609 | 0.663084 | 0.978177 | -6.30063 |
| Adenine | 0.049805 | 7.796649 | 0.666939 | 0.978177 | -6.3029 |
| Glutamate | -0.01401 | 8.715609 | 0.693595 | 0.986676 | -6.31768 |
| Succinate | 0.028008 | 5.383747 | 0.703147 | 0.986676 | -6.32261 |
| sn-Glycero-3-phosphocholine | -0.0363 | 5.70636 | 0.749853 | 0.986676 | -6.34409 |
| Threonine | 0.032615 | 6.07923 | 0.7672 | 0.986676 | -6.351 |
| 4-Aminobutyrate | 0.017233 | 7.872005 | 0.779456 | 0.986676 | -6.35555 |
| Leucine | 0.021774 | 4.85166 | 0.789689 | 0.986676 | -6.35914 |
| Propylene-glycol | 0.027429 | 2.574877 | 0.794821 | 0.986676 | -6.36087 |
| GTP | 0.032404 | 2.680551 | 0.816738 | 0.986676 | -6.36773 |
| Glycine | 0.018052 | 7.561973 | 0.824242 | 0.986676 | -6.36989 |
| Alanine | -0.00897 | 6.72841 | 0.834716 | 0.986676 | -6.37274 |
| IMP | 0.01093 | 2.805823 | 0.860234 | 0.986676 | -6.37892 |
| Phenylalanine | -0.00995 | 4.288332 | 0.893687 | 0.986676 | -6.3854 |
| Glucose-1-Phosphate | -0.02129 | 2.919625 | 0.906876 | 0.986676 | -6.38746 |
| Niacinamide | -0.00748 | 5.838515 | 0.911689 | 0.986676 | -6.38814 |
| Serine | 0.003974 | 6.768895 | 0.911819 | 0.986676 | -6.38816 |
| Isobutyrate | 0.00703 | 2.410397 | 0.915277 | 0.986676 | -6.38863 |
| Valine | 0.004537 | 5.01564 | 0.930412 | 0.986676 | -6.39045 |
| Xanthine | 0.005694 | 3.932873 | 0.946114 | 0.986676 | -6.39196 |
| Hypoxanthine | 0.012031 | 3.424226 | 0.967192 | 0.986676 | -6.39338 |
| Lactate | -0.00058 | 9.299033 | 0.971727 | 0.986676 | -6.39359 |
| Anserine | 0.000964 | 4.954698 | 0.987279 | 0.987279 | -6.39408 |

**Supplementary Table 5:** The results of linear models comparing the mean concentrations of all metabolites between control and severe TBI cases (both 24 hours and 7 days) following the initial injury.

| **Metabolites** | **logFC** | **AveExpr** | **P.Value** | **adj.P.Val** | **B** |
| --- | --- | --- | --- | --- | --- |
| Valine | -0.69152917 | 5.01563969 | 5.14E-26 | 3.39E-24 | 48.53581 |
| Inosine | 0.873268912 | 6.91219303 | 2.34E-25 | 7.71E-24 | 47.02526 |
| Fumarate | 0.669494654 | 5.7854433 | 2.56E-18 | 5.63E-17 | 30.86535 |
| Pyroglutamate | -0.41143647 | 7.23067125 | 3.73E-18 | 6.15E-17 | 30.491 |
| Tyrosine | -0.45867673 | 4.39424205 | 5.23E-18 | 6.9E-17 | 30.15426 |
| Leucine | -0.79442099 | 4.85165998 | 1.39E-17 | 1.53E-16 | 29.17791 |
| Sn-Glycero-3-phosphocholine | 1.074924592 | 5.70635968 | 6.85E-17 | 6.45E-16 | 27.59397 |
| Glucose-1-phosphate | 1.684686228 | 2.91962535 | 1.9E-16 | 1.57E-15 | 26.57694 |
| 3-Hydroxyisobutyrate | -0.4374148 | 2.90047377 | 4.16E-16 | 3.05E-15 | 25.80012 |
| Adenine | 1.002061704 | 7.79664914 | 5.06E-15 | 3.34E-14 | 23.31574 |
| Alanine | -0.36280952 | 6.72841021 | 1.72E-14 | 1.03E-13 | 22.09992 |
| Histidine | 1.113277982 | 5.53765732 | 5.28E-14 | 2.91E-13 | 20.98636 |
| Isoleucine | -0.39642797 | 4.23208092 | 1.94E-13 | 9.84E-13 | 19.69665 |
| Choline | -0.51907583 | 6.02149574 | 2.55E-13 | 1.2E-12 | 19.42352 |
| Glutamate | 0.260653639 | 8.7156094 | 6.75E-12 | 2.97E-11 | 16.1784 |
| Anserine | -0.41128439 | 4.95469821 | 1.08E-10 | 4.47E-10 | 13.43427 |
| 2-Hydroxyisovalerate | -0.41310313 | 3.52794025 | 1.27E-10 | 4.92E-10 | 13.28092 |
| Tryptophan | -0.52460923 | 3.39692074 | 3.12E-09 | 1.14E-08 | 10.12426 |
| Uracil | -0.85911307 | 5.51728167 | 4.89E-09 | 1.7E-08 | 9.680667 |
| O-Phosphocholine | -0.28892011 | 6.43169893 | 7.05E-09 | 2.33E-08 | 9.321506 |
| Phenylalanine | -0.42960669 | 4.28833214 | 1.99E-08 | 6.26E-08 | 8.301591 |
| Acetone | -0.27396278 | 2.80797213 | 1.07E-07 | 3.21E-07 | 6.654858 |
| Glucose | 0.607337593 | 4.38835611 | 1.27E-07 | 3.66E-07 | 6.483959 |
| Citrate | 0.275569513 | 4.7019504 | 1.72E-07 | 4.72E-07 | 6.192695 |
| 4-Aminobutyrate | -0.31122873 | 7.87200503 | 5.83E-07 | 1.49E-06 | 4.99962 |
| Aspartate | -0.18744817 | 8.52058269 | 5.87E-07 | 1.49E-06 | 4.993383 |
| Isobutyrate | -0.31613919 | 2.4103972 | 2E-06 | 4.89E-06 | 3.799331 |
| Acetate | -0.16827733 | 8.83960908 | 2.38E-06 | 5.6E-06 | 3.632775 |
| Ethanolamine | -0.3935091 | 5.72278698 | 4.04E-06 | 9.19E-06 | 3.11932 |
| N-Acetylaspartate | 0.53967205 | 5.91718872 | 4.39E-06 | 9.65E-06 | 3.039066 |
| Xanthine | 0.380601305 | 3.93287281 | 6.41E-06 | 1.37E-05 | 2.671402 |
| ATP | 0.412450063 | 3.37403277 | 1.24E-05 | 2.55E-05 | 2.037323 |
| AMP | -0.18085756 | 3.38118513 | 1.49E-05 | 2.99E-05 | 1.854575 |
| ADP | 0.200580786 | 3.3774816 | 4.4E-05 | 8.53E-05 | 0.816871 |
| Dimethyl-sulfone | -0.24507861 | 2.16167864 | 7.93E-05 | 0.00015 | 0.25201 |
| O-Acetylcarnitine | 0.396315572 | 3.47098433 | 0.000158 | 0.000289 | -0.40467 |
| Glycine | -0.30226702 | 7.56197292 | 0.000164 | 0.000292 | -0.43922 |
| Glycerol | -0.18504997 | 6.94766745 | 0.000347 | 0.000602 | -1.15081 |
| Formate | -0.28144227 | 4.59045768 | 0.00053 | 0.000897 | -1.5514 |
| Creatine | 0.065078328 | 8.77998274 | 0.000674 | 0.001112 | -1.77746 |
| Taurine | -0.09103507 | 8.81147868 | 0.001144 | 0.001842 | -2.27393 |
| Methionine | -0.32774181 | 4.19976733 | 0.001495 | 0.002349 | -2.52335 |
| Propylene-glycol | -0.30912067 | 2.57487709 | 0.002638 | 0.00399 | -3.0503 |
| Hypoxanthine | 0.856636308 | 3.42422593 | 0.00266 | 0.00399 | -3.05798 |
| Urea | -0.22309053 | 5.7140526 | 0.003061 | 0.004489 | -3.18762 |
| GTP | 0.362942533 | 2.68055059 | 0.007474 | 0.010723 | -4.00448 |
| Lactate | 0.04024356 | 9.29903302 | 0.011312 | 0.015885 | -4.37857 |
| UDP-glucose | 0.238907398 | 3.67977419 | 0.017052 | 0.023446 | -4.74494 |
| Dimethylamine | 0.203100747 | 3.04276947 | 0.029434 | 0.039646 | -5.22482 |
| Threonine | -0.22053628 | 6.07922969 | 0.037821 | 0.049923 | -5.44174 |
| Serine | -0.06669229 | 6.76889521 | 0.053578 | 0.069336 | -5.73872 |
| Glutamine | -0.23378359 | 6.75459739 | 0.074665 | 0.094767 | -6.01605 |
| My-Inositol | 0.060716703 | 8.4173641 | 0.079509 | 0.099011 | -6.06787 |
| 3-Methylhistidine | 0.211784321 | 4.55753333 | 0.083156 | 0.101635 | -6.10468 |
| 3-Hydroxybutyrate | -0.15783818 | 4.24557677 | 0.116393 | 0.137644 | -6.3762 |
| Pantothenate | -0.07735006 | 4.0791588 | 0.116789 | 0.137644 | -6.3789 |
| Methanol | 0.037599397 | 7.97244661 | 0.123952 | 0.143524 | -6.42604 |
| Histamine | -0.11671064 | 3.12562888 | 0.133755 | 0.152204 | -6.48585 |
| UDP-galactose | 0.079737823 | 3.29541878 | 0.140102 | 0.156724 | -6.52203 |
| IMP | 0.085399389 | 2.80582284 | 0.15139 | 0.166529 | -6.58204 |
| Carnitine | -0.04594531 | 4.12140061 | 0.334731 | 0.362168 | -7.15233 |
| Acetoacetate | -0.07657295 | 3.89231318 | 0.344862 | 0.367111 | -7.17167 |
| Niacinamide | -0.05896614 | 5.83851477 | 0.360882 | 0.378067 | -7.20072 |
| Pyruvate | 0.077854216 | 4.93076454 | 0.399235 | 0.411711 | -7.26353 |
| Creatinine | 0.063014008 | 5.77285604 | 0.557291 | 0.565865 | -7.44874 |
| Succinate | -0.02483697 | 5.38374682 | 0.72329 | 0.72329 | -7.55953 |

**Supplementary Table 6:** The results of linear model comparing metabolic profile differences between the initial injury of severe TBI (24 hours) and post-injury (7days) of severe TBI.

| **Metabolites** | **logFC** | **AveExpr** | **P.Value** | **adj.P.Val** | **B** |
| --- | --- | --- | --- | --- | --- |
| Dimethylamine | 0.729422682 | 3.042769 | 5.69E-11 | 3.76E-09 | 14.55574 |
| Hypoxanthine | -1.611043436 | 3.424226 | 5.89E-07 | 1.94E-05 | 5.553287 |
| Glucose | -0.567329325 | 4.388356 | 4.16E-06 | 9.14E-05 | 3.669101 |
| N-Acetylaspartate | 0.560296612 | 5.917189 | 1.11E-05 | 0.000183 | 2.729426 |
| 3-Methylhistidine | -0.581735214 | 4.557533 | 2.45E-05 | 0.000323 | 1.970904 |
| Valine | 0.21303259 | 5.01564 | 0.000138 | 0.001518 | 0.326511 |
| Creatine | 0.073339647 | 8.779983 | 0.000446 | 0.004209 | -0.77846 |
| Carnitine | 0.183523347 | 4.121401 | 0.000565 | 0.004663 | -0.99945 |
| Leucine | 0.285803103 | 4.85166 | 0.001024 | 0.007507 | -1.55308 |
| 4-Aminobutyrate | 0.189863279 | 7.872005 | 0.003615 | 0.023862 | -2.71634 |
| Creatinine | -0.326322568 | 5.772856 | 0.006076 | 0.036454 | -3.18829 |
| Taurine | -0.082137438 | 8.811479 | 0.006644 | 0.036541 | -3.2691 |
| Glycerol | 0.140637775 | 6.947667 | 0.011288 | 0.057309 | -3.74494 |
| Tyrosine | 0.120134095 | 4.394242 | 0.013835 | 0.065224 | -3.92594 |
| Threonine | -0.273203695 | 6.07923 | 0.018523 | 0.081503 | -4.18364 |
| Histidine | -0.298861479 | 5.537657 | 0.035378 | 0.145934 | -4.74581 |
| AMP | -0.09132345 | 3.381185 | 0.037874 | 0.147042 | -4.80418 |
| Methionine | 0.22662085 | 4.199767 | 0.040976 | 0.150245 | -4.87131 |
| IMP | -0.128982885 | 2.805823 | 0.047647 | 0.165511 | -4.99923 |
| ADP | -0.101115677 | 3.377482 | 0.051319 | 0.169352 | -5.0618 |
| Isoleucine | 0.097197961 | 4.232081 | 0.061506 | 0.193253 | -5.21329 |
| Xanthine | 0.163274411 | 3.932873 | 0.064418 | 0.193253 | -5.25171 |
| Anserine | 0.109780465 | 4.954698 | 0.082787 | 0.234399 | -5.45795 |
| Sn-Glycero-3-phosphocholine | 0.20508154 | 5.70636 | 0.085236 | 0.234399 | -5.48166 |
| Glutamine | -0.226442809 | 6.754597 | 0.112429 | 0.287235 | -5.70393 |
| Methanol | 0.042191532 | 7.972447 | 0.113153 | 0.287235 | -5.70901 |
| Urea | 0.126482212 | 5.714053 | 0.117754 | 0.287844 | -5.74051 |
| Dimethyl-sulfone | 0.098844894 | 2.161679 | 0.131706 | 0.310451 | -5.82826 |
| Formate | -0.125257179 | 4.590458 | 0.147377 | 0.335411 | -5.91523 |
| Phenylalanine | 0.106904562 | 4.288332 | 0.169011 | 0.371825 | -6.01949 |
| Tryptophan | 0.115781571 | 3.396921 | 0.193687 | 0.4009 | -6.12117 |
| UDP-galactose | 0.076314679 | 3.295419 | 0.194376 | 0.4009 | -6.12378 |
| Ethanolamine | 0.11242552 | 5.722787 | 0.20548 | 0.41096 | -6.16456 |
| Propylene-glycol | 0.135293359 | 2.574877 | 0.21886 | 0.417623 | -6.21036 |
| Pyroglutamate | -0.052271222 | 7.230671 | 0.224652 | 0.417623 | -6.22917 |
| O-Acetylcarnitine | 0.133736796 | 3.470984 | 0.227794 | 0.417623 | -6.23913 |
| 3-Hydroxyisobutyrate | 0.057724056 | 2.900474 | 0.248669 | 0.443571 | -6.30134 |
| Inosine | 0.07907826 | 6.912193 | 0.258121 | 0.448316 | -6.32746 |
| Aspartate | -0.042121008 | 8.520583 | 0.275768 | 0.466684 | -6.37317 |
| Succinate | 0.080768221 | 5.383747 | 0.291629 | 0.481189 | -6.41123 |
| 2-Hydroxyisovalerate | -0.058216725 | 3.52794 | 0.359426 | 0.562603 | -6.54801 |
| Histamine | -0.076613856 | 3.125629 | 0.364633 | 0.562603 | -6.55706 |
| Pantothenate | -0.048326444 | 4.079159 | 0.366544 | 0.562603 | -6.56034 |
| Isobutyrate | -0.060145151 | 2.410397 | 0.382184 | 0.57209 | -6.58632 |
| O-Phosphocholine | 0.043226655 | 6.431699 | 0.390061 | 0.57209 | -6.59883 |
| 3-Hydroxybutyrate | 0.091337632 | 4.245577 | 0.402322 | 0.577245 | -6.61762 |
| Fumarate | 0.053071641 | 5.785443 | 0.444634 | 0.604534 | -6.67645 |
| Glutamate | -0.028173933 | 8.715609 | 0.446505 | 0.604534 | -6.67886 |
| Adenine | -0.091217491 | 7.796649 | 0.448821 | 0.604534 | -6.68181 |
| Uracil | -0.105013283 | 5.517282 | 0.47694 | 0.624185 | -6.71587 |
| Niacinamide | 0.049353281 | 5.838515 | 0.482324 | 0.624185 | -6.72203 |
| Choline | 0.044504276 | 6.021496 | 0.512897 | 0.650984 | -6.75492 |
| ATP | 0.053948019 | 3.374033 | 0.583335 | 0.726417 | -6.81888 |
| Acetone | 0.023795788 | 2.807972 | 0.650722 | 0.783107 | -6.86706 |
| Sucrose | 0.085206505 | 2.919625 | 0.652589 | 0.783107 | -6.86824 |
| Acetate | -0.014437005 | 8.839609 | 0.695475 | 0.811049 | -6.89308 |
| UDP-glucose | 0.041432663 | 3.679774 | 0.700451 | 0.811049 | -6.8957 |
| Alanine | -0.013006489 | 6.72841 | 0.771126 | 0.876726 | -6.92755 |
| Pyruvate | 0.027570744 | 4.930765 | 0.78374 | 0.876726 | -6.93223 |
| Myo-Inositol | 0.008460638 | 8.417364 | 0.82129 | 0.898404 | -6.94444 |
| Serine | 0.007995944 | 6.768895 | 0.830343 | 0.898404 | -6.94701 |
| Glycine | -0.010776762 | 7.561973 | 0.898526 | 0.956353 | -6.96191 |
| GTP | 0.015903876 | 2.680551 | 0.912883 | 0.956353 | -6.96407 |
| Acetoacetate | 0.007001092 | 3.892313 | 0.936679 | 0.96595 | -6.96691 |
| Citrate | -0.002636824 | 4.70195 | 0.960936 | 0.97572 | -6.96888 |
| Lactate | 0.000348576 | 9.299033 | 0.983691 | 0.983691 | -6.96987 |

**Supplementary Table 7:** The results of linear model comparing metabolic profile between all severe TBI cases (both 24 hours and 7 days) with all mild TBI (both 24 hours and 7 days).

| **Metabolites** | **logFC** | **AveExpr** | **P.Value** | **adj.P.Val** | **B** |
| --- | --- | --- | --- | --- | --- |
| Glucose-1-phosphate | 1.751457514 | 2.919625 | 4.85E-24 | 3.2E-22 | 44.03667 |
| Dimethylamine | 0.717574574 | 3.042769 | 2.8E-17 | 9.23E-16 | 28.53373 |
| N-Acetylaspartate | 0.782742344 | 5.917189 | 5.63E-15 | 1.24E-13 | 23.26285 |
| Creatinine | -0.611359185 | 5.772856 | 3.22E-11 | 5.31E-10 | 14.69068 |
| Carnitine | -0.255340066 | 4.121401 | 2.62E-10 | 3.45E-09 | 12.62315 |
| ATP | -0.462888994 | 3.374033 | 1.21E-09 | 1.18E-08 | 11.11814 |
| Creatine | 0.09547556 | 8.779983 | 1.25E-09 | 1.18E-08 | 11.08071 |
| ADP | -0.231737353 | 3.377482 | 4.78E-09 | 3.95E-08 | 9.762 |
| Formate | -0.35869165 | 4.590458 | 4.4E-08 | 3.22E-07 | 7.585914 |
| GTP | -0.590742987 | 2.680551 | 8.66E-08 | 5.72E-07 | 6.922129 |
| Taurine | -0.119163652 | 8.811479 | 1.25E-07 | 7.53E-07 | 6.559883 |
| 3-Hydroxybutyrate | -0.383334712 | 4.245577 | 2.48E-06 | 1.27E-05 | 3.653835 |
| IMP | -0.22708649 | 2.805823 | 2.51E-06 | 1.27E-05 | 3.64342 |
| Pyroglutamate | -0.144429083 | 7.230671 | 6.12E-06 | 2.88E-05 | 2.779858 |
| Leucine | 0.28234124 | 4.85166 | 7.69E-06 | 3.38E-05 | 2.558228 |
| Serine | -0.121463709 | 6.768895 | 1.16E-05 | 4.78E-05 | 2.163633 |
| Uracil | -0.446774392 | 5.517282 | 4.05E-05 | 0.000157 | 0.958831 |
| Succinate | -0.202429601 | 5.383747 | 0.000292 | 0.001072 | -0.92517 |
| AMP | -0.106622081 | 3.381185 | 0.000776 | 0.002697 | -1.84619 |
| 3-Methylhistidine | -0.30481057 | 4.557533 | 0.001506 | 0.004968 | -2.46525 |
| Xanthine | 0.192911584 | 3.932873 | 0.002381 | 0.007008 | -2.89094 |
| Tyrosine | 0.105458195 | 4.394242 | 0.002498 | 0.007008 | -2.93518 |
| Fumarate | 0.151745879 | 5.785443 | 0.00253 | 0.007008 | -2.94678 |
| Histidine | -0.307458233 | 5.537657 | 0.002548 | 0.007008 | -2.95359 |
| Valine | 0.117086041 | 5.01564 | 0.002775 | 0.007325 | -3.03221 |
| Glucose | 0.237264116 | 4.388356 | 0.005105 | 0.012958 | -3.59226 |
| Ethanolamine | 0.177509742 | 5.722787 | 0.005475 | 0.013266 | -3.65624 |
| 4-Aminobutyrate | 0.12793361 | 7.872005 | 0.005628 | 0.013266 | -3.6814 |
| Hypoxanthine | 0.604079385 | 3.424226 | 0.005948 | 0.013536 | -3.7317 |
| Niacinamide | -0.136279421 | 5.838515 | 0.007109 | 0.015641 | -3.89397 |
| Methionine | 0.196227662 | 4.199767 | 0.01304 | 0.027762 | -4.44058 |
| Urea | -0.142063286 | 5.714053 | 0.01406 | 0.028999 | -4.50786 |
| Acetate | -0.059405771 | 8.839609 | 0.024798 | 0.049596 | -5.00964 |
| Citrate | -0.083859793 | 4.70195 | 0.02989 | 0.058022 | -5.17258 |
| O-Phosphocholine | -0.074337458 | 6.431699 | 0.038804 | 0.072064 | -5.39812 |
| Choline | 0.100341507 | 6.021496 | 0.039308 | 0.072064 | -5.40919 |
| Adenine | -0.169721412 | 7.796649 | 0.048729 | 0.086923 | -5.59263 |
| Alanine | -0.058098483 | 6.72841 | 0.069093 | 0.120003 | -5.88593 |
| Phenylalanine | 0.099007723 | 4.288332 | 0.07348 | 0.124351 | -5.93696 |
| Dimethyl-sulfone | -0.08207107 | 2.161679 | 0.0783 | 0.129195 | -5.98937 |
| Anserine | 0.077156532 | 4.954698 | 0.085686 | 0.137934 | -6.06329 |
| Threonine | -0.125123768 | 6.07923 | 0.12565 | 0.19745 | -6.37074 |
| Inosine | 0.074961105 | 6.912193 | 0.131717 | 0.20217 | -6.40779 |
| Glutamate | 0.038472574 | 8.715609 | 0.144329 | 0.216494 | -6.47906 |
| UDP-galactose | 0.058787244 | 3.295419 | 0.159133 | 0.233395 | -6.55425 |
| Acetoacetate | -0.086126731 | 3.892313 | 0.170225 | 0.244236 | -6.60556 |
| Acetone | 0.044474594 | 2.807972 | 0.234311 | 0.329032 | -6.84138 |
| O-Acetylcarnitine | -0.090985106 | 3.470984 | 0.24737 | 0.333833 | -6.88 |
| Glycerol | 0.044976862 | 6.947667 | 0.247846 | 0.333833 | -6.88136 |
| Tryptophan | 0.070436705 | 3.396921 | 0.264598 | 0.34927 | -6.9273 |
| Aspartate | -0.029775628 | 8.520583 | 0.277528 | 0.359153 | -6.96036 |
| Glutamine | -0.107108309 | 6.754597 | 0.288394 | 0.366038 | -6.98667 |
| Histamine | 0.060560082 | 3.125629 | 0.312718 | 0.389423 | -7.04124 |
| Sn-Glycero-3-phosphocholine | -0.078225821 | 5.70636 | 0.352491 | 0.430822 | -7.11945 |
| Isoleucine | -0.03130027 | 4.232081 | 0.393038 | 0.471646 | -7.18767 |
| 2-Hydroxyisovalerate | 0.035966275 | 3.52794 | 0.424658 | 0.50049 | -7.23425 |
| Glycine | 0.044827854 | 7.561973 | 0.455247 | 0.527128 | -7.27461 |
| Pyruvate | -0.05055173 | 4.930765 | 0.478616 | 0.544632 | -7.30269 |
| Isobutyrate | -0.03205011 | 2.410397 | 0.511291 | 0.571953 | -7.33838 |
| 3-Hydroxyisobutyrate | -0.021986113 | 2.900474 | 0.534845 | 0.588329 | -7.36176 |
| Methanol | 0.01008237 | 7.972447 | 0.591745 | 0.640249 | -7.41108 |
| Myo-inositol | 0.013667455 | 8.417364 | 0.607223 | 0.646399 | -7.42291 |
| Pantothenate | -0.018367205 | 4.079159 | 0.628145 | 0.658056 | -7.4379 |
| Propylene-glycol | 0.035391667 | 2.574877 | 0.649325 | 0.669616 | -7.45197 |
| Lactate | 0.001945377 | 9.299033 | 0.872263 | 0.885682 | -7.54343 |
| UDP-glucose | 0.000906875 | 3.679774 | 0.990528 | 0.990528 | -7.55643 |

**Supplementary Table 8:** The results of metabolic set enrichment analysis based on the metabolic profile differences between initial mild TBI injury (24 hours) with post mild TBI injury (7 days)

| **Pathway** | **P.Value** | **adj.P.Val** | **ES** | **NES** | **Observed_Hits** |
| --- | --- | --- | --- | --- | --- |
| 2-Oxocarboxylic acid metabolism | 0.85944 | 0.96982 | -0.38637 | -0.66919 | 6 |
| Alanine, aspartate and glutamate metabolism | 0.08420 | 0.96982 | 0.70497 | 1.39810 | 10 |
| Amino sugar and nucleotide sugar metabolism | 0.40796 | 0.96982 | -0.76563 | -1.10267 | 2 |
| Aminoacyl-tRNA biosynthesis | 0.96982 | 0.96982 | -0.27236 | -0.57313 | 14 |
| Arginine and proline metabolism | 0.04219 | 0.96982 | 0.88755 | 1.44062 | 4 |
| Arginine biosynthesis | 0.70220 | 0.96982 | 0.47998 | 0.81355 | 5 |
| Ascorbate and aldarate metabolism | 0.91065 | 0.96982 | 0.44997 | 0.64353 | 2 |
| Biosynthesis of amino acids | 0.95880 | 0.96982 | 0.27636 | 0.59735 | 14 |
| Biosynthesis of cofactors | 0.77046 | 0.96982 | 0.37225 | 0.79038 | 13 |
| Biosynthesis of nucleotide sugars | 0.95296 | 0.96982 | 0.32795 | 0.50277 | 3 |
| Butanoate metabolism | 0.75025 | 0.96982 | 0.47671 | 0.77377 | 4 |
| Carbon metabolism | 0.76249 | 0.96982 | 0.38859 | 0.79118 | 11 |
| Circadian entrainment | 0.68776 | 0.96982 | -0.66154 | -0.88119 | 1 |
| Citrate cycle (TCA cycle) | 0.12096 | 0.96982 | 0.83215 | 1.35069 | 4 |
| Cocaine addiction | 0.68776 | 0.96982 | -0.66154 | -0.88119 | 1 |
| Cysteine and methionine metabolism | 0.79245 | 0.96982 | 0.54799 | 0.78371 | 2 |
| Ether lipid metabolism | 0.71929 | 0.96982 | -0.64615 | -0.86070 | 1 |
| Folate biosynthesis | 0.87961 | 0.96982 | 0.56923 | 0.75589 | 1 |
| FoxO signaling pathway | 0.68776 | 0.96982 | -0.66154 | -0.88119 | 1 |
| GABAergic synapse | 0.78893 | 0.96982 | 0.61538 | 0.81718 | 1 |
| Galactose metabolism | 0.84057 | 0.96982 | -0.40798 | -0.68062 | 5 |
| Gastric acid secretion | 0.54680 | 0.96982 | 0.73846 | 0.98061 | 1 |
| Glutamatergic synapse | 0.47282 | 0.96982 | 0.73553 | 1.05193 | 2 |
| Glutathione metabolism | 0.45390 | 0.96982 | -0.70855 | -1.07354 | 3 |
| Glycerolipid metabolism | 0.41426 | 0.96982 | -0.80000 | -1.06563 | 1 |
| Glycerophospholipid metabolism | 0.10630 | 0.96982 | -0.84515 | -1.34740 | 4 |
| Glycine, serine and threonine metabolism | 0.65124 | 0.96982 | 0.51080 | 0.86580 | 5 |
| Glycolysis / Gluconeogenesis | 0.45248 | 0.96982 | -0.67397 | -1.07449 | 4 |
| Glyoxylate and dicarboxylate metabolism | 0.42987 | 0.96982 | 0.58305 | 1.06548 | 7 |
| Histidine metabolism | 0.94778 | 0.96982 | -0.30916 | -0.53546 | 6 |
| Huntington disease | 0.68776 | 0.96982 | -0.66154 | -0.88119 | 1 |
| Inflammatory mediator regulation of TRP channels | 0.13461 | 0.96982 | 0.90121 | 1.28887 | 2 |
| Inositol phosphate metabolism | 0.23495 | 0.96982 | 0.89231 | 1.18491 | 1 |
| Long-term potentiation | 0.68776 | 0.96982 | -0.66154 | -0.88119 | 1 |
| Longevity regulating pathway | 0.63273 | 0.96982 | -0.69231 | -0.92218 | 1 |
| Melanogenesis | 0.63748 | 0.96982 | 0.69231 | 0.91933 | 1 |
| Metabolic pathways | 0.91707 | 0.96982 | 0.28376 | 0.74819 | 51 |
| Morphine addiction | 0.78893 | 0.96982 | 0.61538 | 0.81718 | 1 |
| Nicotinate and nicotinamide metabolism | 0.24086 | 0.96982 | -0.84432 | -1.21601 | 2 |
| Nitrogen metabolism | 0.47282 | 0.96982 | 0.73553 | 1.05193 | 2 |
| Pantothenate and CoA biosynthesis | 0.23730 | 0.96982 | -0.77615 | -1.23740 | 4 |
| Pathways in cancer | 0.09379 | 0.96982 | 0.96923 | 1.28706 | 1 |
| Pathways of neurodegeneration - multiple diseases | 0.68776 | 0.96982 | -0.66154 | -0.88119 | 1 |
| Pentose and glucuronate interconversions | 0.26917 | 0.96982 | -0.87692 | -1.16809 | 1 |
| Phenylalanine metabolism | 0.82645 | 0.96982 | 0.52748 | 0.75437 | 2 |
| Phenylalanine, tyrosine and tryptophan biosynthesis | 0.82645 | 0.96982 | 0.52748 | 0.75437 | 2 |
| Phosphatidylinositol signaling system | 0.23495 | 0.96982 | 0.89231 | 1.18491 | 1 |
| Phospholipase D signaling pathway | 0.68776 | 0.96982 | -0.66154 | -0.88119 | 1 |
| Platelet activation | 0.35612 | 0.96982 | 0.83077 | 1.10319 | 1 |
| Porphyrin and chlorophyll metabolism | 0.89007 | 0.96982 | -0.45313 | -0.65260 | 2 |
| Primary bile acid biosynthesis | 0.74875 | 0.96982 | -0.56944 | -0.82011 | 2 |
| Propanoate metabolism | 0.72621 | 0.96982 | 0.64615 | 0.85804 | 1 |
| Purine metabolism | 0.44484 | 0.96982 | 0.51808 | 1.02746 | 10 |
| Pyrimidine metabolism | 0.83001 | 0.96982 | 0.51674 | 0.73902 | 2 |
| Pyruvate metabolism | 0.03617 | 0.96982 | 0.89489 | 1.45253 | 4 |
| Ras signaling pathway | 0.87961 | 0.96982 | 0.56923 | 0.75589 | 1 |
| Retrograde endocannabinoid signaling | 0.68776 | 0.96982 | -0.66154 | -0.88119 | 1 |
| Selenocompound metabolism | 0.75289 | 0.96982 | -0.63077 | -0.84021 | 1 |
| Spinocerebellar ataxia | 0.68776 | 0.96982 | -0.66154 | -0.88119 | 1 |
| Starch and sucrose metabolism | 0.28283 | 0.96982 | -0.82541 | -1.18878 | 2 |
| Taste transduction | 0.69598 | 0.96982 | -0.60938 | -0.87764 | 2 |
| Taurine and hypotaurine metabolism | 0.53298 | 0.96982 | -0.73846 | -0.98366 | 1 |
| Tryptophan metabolism | 0.38252 | 0.96982 | -0.81538 | -1.08612 | 1 |
| Tyrosine metabolism | 0.19630 | 0.96982 | 0.79118 | 1.28419 | 4 |
| Ubiquinone and other terpenoid-quinone biosynthesis | 0.63748 | 0.96982 | 0.69231 | 0.91933 | 1 |
| Valine, leucine and isoleucine biosynthesis | 0.88737 | 0.96982 | -0.38710 | -0.61714 | 4 |
| Valine, leucine and isoleucine degradation | 0.86498 | 0.96982 | 0.39594 | 0.64266 | 4 |
| beta-Alanine metabolism | 0.63960 | 0.96982 | -0.56428 | -0.89961 | 4 |
| cAMP signaling pathway | 0.66693 | 0.96982 | 0.57352 | 0.87925 | 3 |
| mTOR signaling pathway | 0.63273 | 0.96982 | -0.69231 | -0.92218 | 1 |

**Supplementary Table 9:** The results of metabolic set enrichment analysis based on the metabolic profile differences between initial severe TBI injury (24 hours) with post severe TBI injury (7 days)

| **Pathway** | **P.Value** | **adj.P.Val** | **ES** | **NES** | **Observed_Hits** |
| --- | --- | --- | --- | --- | --- |
| 2-Oxocarboxylic acid metabolism | 0.12637 | 0.85057 | 0.76353 | 1.34133 | 6 |
| Alanine, aspartate and glutamate metabolism | 0.23143 | 0.85057 | 0.62343 | 1.22574 | 10 |
| Amino sugar and nucleotide sugar metabolism | 0.85307 | 0.92015 | 0.52457 | 0.74915 | 2 |
| Aminoacyl-tRNA biosynthesis | 0.37212 | 0.85057 | 0.51301 | 1.08128 | 14 |
| Arginine and proline metabolism | 0.08698 | 0.85057 | 0.84637 | 1.36607 | 4 |
| Arginine biosynthesis | 0.89899 | 0.92015 | -0.32971 | -0.59184 | 5 |
| Ascorbate and aldarate metabolism | 0.75218 | 0.92015 | -0.53125 | -0.77959 | 2 |
| Biosynthesis of amino acids | 0.19024 | 0.85057 | 0.59341 | 1.25073 | 14 |
| Biosynthesis of cofactors | 0.90700 | 0.92015 | 0.30771 | 0.64089 | 13 |
| Biosynthesis of nucleotide sugars | 0.87168 | 0.92015 | 0.46032 | 0.70024 | 3 |
| Butanoate metabolism | 0.48488 | 0.85057 | 0.63092 | 1.01832 | 4 |
| Carbon metabolism | 0.12165 | 0.85057 | -0.61867 | -1.35551 | 11 |
| Circadian entrainment | 0.57461 | 0.85057 | -0.72308 | -0.95834 | 1 |
| Citrate cycle (TCA cycle) | 0.82835 | 0.92015 | 0.45061 | 0.72730 | 4 |
| Cocaine addiction | 0.57461 | 0.85057 | -0.72308 | -0.95834 | 1 |
| Cysteine and methionine metabolism | 0.33781 | 0.85057 | 0.78851 | 1.12607 | 2 |
| Ether lipid metabolism | 0.42987 | 0.85057 | 0.80000 | 1.05783 | 1 |
| Folate biosynthesis | 0.84641 | 0.92015 | -0.58462 | -0.77483 | 1 |
| FoxO signaling pathway | 0.57461 | 0.85057 | -0.72308 | -0.95834 | 1 |
| GABAergic synapse | 0.22318 | 0.85057 | 0.90769 | 1.20023 | 1 |
| Galactose metabolism | 0.56124 | 0.85057 | 0.56696 | 0.95851 | 5 |
| Gastric acid secretion | 0.48480 | 0.85057 | -0.76923 | -1.01951 | 1 |
| Glutamatergic synapse | 0.43854 | 0.85057 | -0.73438 | -1.07767 | 2 |
| Glutathione metabolism | 0.42332 | 0.85057 | -0.68570 | -1.09201 | 3 |
| Glycerolipid metabolism | 0.25196 | 0.85057 | 0.89231 | 1.17989 | 1 |
| Glycerophospholipid metabolism | 0.62878 | 0.89826 | 0.56452 | 0.91114 | 4 |
| Glycine, serine and threonine metabolism | 0.58324 | 0.85057 | 0.55722 | 0.94204 | 5 |
| Glycolysis / Gluconeogenesis | 0.00752 | 0.52632 | -0.93598 | -1.59459 | 4 |
| Glyoxylate and dicarboxylate metabolism | 0.24356 | 0.85057 | -0.63491 | -1.23492 | 7 |
| Histidine metabolism | 0.28924 | 0.85057 | -0.63926 | -1.19888 | 6 |
| Huntington disease | 0.57461 | 0.85057 | -0.72308 | -0.95834 | 1 |
| Inflammatory mediator regulation of TRP channels | 0.82999 | 0.92015 | -0.48438 | -0.71080 | 2 |
| Inositol phosphate metabolism | 0.90682 | 0.92015 | -0.55385 | -0.73404 | 1 |
| Long-term potentiation | 0.57461 | 0.85057 | -0.72308 | -0.95834 | 1 |
| Longevity regulating pathway | 0.25055 | 0.85057 | -0.89231 | -1.18263 | 1 |
| Melanogenesis | 0.28577 | 0.85057 | 0.87692 | 1.15955 | 1 |
| Metabolic pathways | 0.77763 | 0.92015 | -0.30021 | -0.86369 | 51 |
| Morphine addiction | 0.22318 | 0.85057 | 0.90769 | 1.20023 | 1 |
| Nicotinate and nicotinamide metabolism | 0.87707 | 0.92015 | -0.45313 | -0.66495 | 2 |
| Nitrogen metabolism | 0.43854 | 0.85057 | -0.73438 | -1.07767 | 2 |
| Pantothenate and CoA biosynthesis | 0.31626 | 0.85057 | 0.71337 | 1.15139 | 4 |
| Pathways in cancer | 0.87764 | 0.92015 | 0.56923 | 0.75269 | 1 |
| Pathways of neurodegeneration - multiple diseases | 0.57461 | 0.85057 | -0.72308 | -0.95834 | 1 |
| Pentose and glucuronate interconversions | 0.96702 | 0.96702 | -0.52308 | -0.69326 | 1 |
| Phenylalanine metabolism | 0.41743 | 0.85057 | 0.75000 | 1.07108 | 2 |
| Phenylalanine, tyrosine and tryptophan biosynthesis | 0.41743 | 0.85057 | 0.75000 | 1.07108 | 2 |
| Phosphatidylinositol signaling system | 0.90682 | 0.92015 | -0.55385 | -0.73404 | 1 |
| Phospholipase D signaling pathway | 0.57461 | 0.85057 | -0.72308 | -0.95834 | 1 |
| Platelet activation | 0.31194 | 0.85057 | -0.86154 | -1.14185 | 1 |
| Porphyrin and chlorophyll metabolism | 0.57912 | 0.85057 | -0.65625 | -0.96302 | 2 |
| Primary bile acid biosynthesis | 0.10375 | 0.85057 | -0.91661 | -1.34509 | 2 |
| Propanoate metabolism | 0.78688 | 0.92015 | 0.61538 | 0.81372 | 1 |
| Purine metabolism | 0.14347 | 0.85057 | -0.62313 | -1.32618 | 10 |
| Pyrimidine metabolism | 0.50174 | 0.85057 | -0.70313 | -1.03181 | 2 |
| Pyruvate metabolism | 0.72910 | 0.92015 | -0.45161 | -0.76939 | 4 |
| Ras signaling pathway | 0.84641 | 0.92015 | -0.58462 | -0.77483 | 1 |
| Retrograde endocannabinoid signaling | 0.57461 | 0.85057 | -0.72308 | -0.95834 | 1 |
| Selenocompound metabolism | 0.69263 | 0.92015 | -0.66154 | -0.87678 | 1 |
| Spinocerebellar ataxia | 0.57461 | 0.85057 | -0.72308 | -0.95834 | 1 |
| Starch and sucrose metabolism | 0.78008 | 0.92015 | -0.51563 | -0.75666 | 2 |
| Taste transduction | 0.78008 | 0.92015 | -0.51563 | -0.75666 | 2 |
| Taurine and hypotaurine metabolism | 0.16193 | 0.85057 | -0.93846 | -1.24380 | 1 |
| Tryptophan metabolism | 0.58080 | 0.85057 | 0.72308 | 0.95612 | 1 |
| Tyrosine metabolism | 0.41320 | 0.85057 | 0.66364 | 1.07113 | 4 |
| Ubiquinone and other terpenoid-quinone biosynthesis | 0.28577 | 0.85057 | 0.87692 | 1.15955 | 1 |
| Valine, leucine and isoleucine biosynthesis | 0.35201 | 0.85057 | 0.69408 | 1.12027 | 4 |
| Valine, leucine and isoleucine degradation | 0.06190 | 0.85057 | 0.86746 | 1.40010 | 4 |
| beta-Alanine metabolism | 0.82206 | 0.92015 | -0.39536 | -0.67357 | 4 |
| cAMP signaling pathway | 0.38004 | 0.85057 | 0.72303 | 1.09989 | 3 |
| mTOR signaling pathway | 0.25055 | 0.85057 | -0.89231 | -1.18263 | 1 |

**Supplementary Table 10:** The results of metabolic set enrichment analysis based on the metabolic profile differences between mild TBI (both 24 hours and 7 days) with severe TBI (both 24 hours and 7 days).

| **Pathway** | **P.Value** | **adj.P.Val** | **ES** | **NES** | **Observed_Hits** |
| --- | --- | --- | --- | --- | --- |
| 2-Oxocarboxylic acid metabolism | 0.46359 | 0.90760 | 0.58079 | 1.03388 | 6 |
| Alanine, aspartate and glutamate metabolism | 0.40145 | 0.90760 | 0.53186 | 1.06137 | 10 |
| Amino sugar and nucleotide sugar metabolism | 0.49552 | 0.90760 | 0.69797 | 1.03302 | 2 |
| Aminoacyl-tRNA biosynthesis | 0.62235 | 0.90760 | 0.41634 | 0.89598 | 14 |
| Arginine and proline metabolism | 0.26422 | 0.90760 | 0.74445 | 1.23526 | 4 |
| Arginine biosynthesis | 0.94415 | 0.96679 | 0.29189 | 0.50486 | 5 |
| Ascorbate and aldarate metabolism | 0.71631 | 0.93086 | 0.55940 | 0.82793 | 2 |
| Biosynthesis of amino acids | 0.47341 | 0.90760 | 0.46080 | 0.99167 | 14 |
| Biosynthesis of cofactors | 0.10319 | 0.90760 | -0.63940 | -1.34723 | 13 |
| Biosynthesis of nucleotide sugars | 0.60613 | 0.90760 | 0.58325 | 0.92145 | 3 |
| Butanoate metabolism | 0.98403 | 0.98403 | -0.28447 | -0.46023 | 4 |
| Carbon metabolism | 0.83972 | 0.96679 | -0.35876 | -0.73061 | 11 |
| Circadian entrainment | 0.57762 | 0.90760 | 0.72308 | 0.95790 | 1 |
| Citrate cycle (TCA cycle) | 0.95298 | 0.96679 | -0.34394 | -0.55645 | 4 |
| Cocaine addiction | 0.57762 | 0.90760 | 0.72308 | 0.95790 | 1 |
| Cysteine and methionine metabolism | 0.57446 | 0.90760 | 0.65173 | 0.96457 | 2 |
| Ether lipid metabolism | 0.91001 | 0.96679 | -0.55385 | -0.73475 | 1 |
| Folate biosynthesis | 0.18018 | 0.90760 | -0.92308 | -1.22459 | 1 |
| FoxO signaling pathway | 0.57762 | 0.90760 | 0.72308 | 0.95790 | 1 |
| GABAergic synapse | 0.36642 | 0.90760 | 0.83077 | 1.10056 | 1 |
| Galactose metabolism | 0.00377 | 0.13200 | 0.93485 | 1.61694 | 5 |
| Gastric acid secretion | 0.73146 | 0.93086 | 0.64615 | 0.85599 | 1 |
| Glutamatergic synapse | 0.87940 | 0.96679 | 0.43750 | 0.64751 | 2 |
| Glutathione metabolism | 0.50364 | 0.90760 | -0.67228 | -1.02541 | 3 |
| Glycerolipid metabolism | 0.67471 | 0.93086 | 0.67692 | 0.89675 | 1 |
| Glycerophospholipid metabolism | 0.00205 | 0.13200 | 0.99982 | 1.47976 | 4 |
| Glycine, serine and threonine metabolism | 0.28473 | 0.90760 | 0.70078 | 1.21209 | 5 |
| Glycolysis / Gluconeogenesis | 0.83101 | 0.96679 | 0.39074 | 0.64836 | 4 |
| Glyoxylate and dicarboxylate metabolism | 0.49283 | 0.90760 | -0.54885 | -1.00959 | 7 |
| Histidine metabolism | 0.82310 | 0.96679 | -0.41256 | -0.73194 | 6 |
| Huntington disease | 0.57762 | 0.90760 | 0.72308 | 0.95790 | 1 |
| Inflammatory mediator regulation of TRP channels | 0.07719 | 0.90760 | -0.91518 | -1.30878 | 2 |
| Inositol phosphate metabolism | 0.84952 | 0.96679 | 0.58462 | 0.77447 | 1 |
| Long-term potentiation | 0.57762 | 0.90760 | 0.72308 | 0.95790 | 1 |
| Longevity regulating pathway | 0.41881 | 0.90760 | -0.80000 | -1.06131 | 1 |
| Melanogenesis | 0.21376 | 0.90760 | 0.90769 | 1.20247 | 1 |
| Metabolic pathways | 0.18336 | 0.90760 | 0.43895 | 1.15709 | 51 |
| Morphine addiction | 0.36642 | 0.90760 | 0.83077 | 1.10056 | 1 |
| Nicotinate and nicotinamide metabolism | 0.74233 | 0.93086 | -0.59375 | -0.84911 | 2 |
| Nitrogen metabolism | 0.87940 | 0.96679 | 0.43750 | 0.64751 | 2 |
| Pantothenate and CoA biosynthesis | 0.90366 | 0.96679 | -0.39245 | -0.63492 | 4 |
| Pathways in cancer | 0.24698 | 0.90760 | 0.89231 | 1.18208 | 1 |
| Pathways of neurodegeneration - multiple diseases | 0.57762 | 0.90760 | 0.72308 | 0.95790 | 1 |
| Pentose and glucuronate interconversions | 0.93949 | 0.96679 | 0.53846 | 0.71333 | 1 |
| Phenylalanine metabolism | 0.32472 | 0.90760 | 0.78125 | 1.15627 | 2 |
| Phenylalanine, tyrosine and tryptophan biosynthesis | 0.32472 | 0.90760 | 0.78125 | 1.15627 | 2 |
| Phosphatidylinositol signaling system | 0.84952 | 0.96679 | 0.58462 | 0.77447 | 1 |
| Phospholipase D signaling pathway | 0.57762 | 0.90760 | 0.72308 | 0.95790 | 1 |
| Platelet activation | 0.12174 | 0.90760 | -0.95385 | -1.26541 | 1 |
| Porphyrin and chlorophyll metabolism | 0.62133 | 0.90760 | 0.62500 | 0.92501 | 2 |
| Primary bile acid biosynthesis | 0.018391 | 0.90760 | -0.85907 | -1.22853 | 2 |
| Propanoate metabolism | 0.38928 | 0.90760 | -0.81538 | -1.08172 | 1 |
| Purine metabolism | 0.23931 | 0.90760 | -0.60599 | -1.20548 | 10 |
| Pyrimidine metabolism | 0.50124 | 0.90760 | -0.71864 | -1.02772 | 2 |
| Pyruvate metabolism | 0.74401 | 0.93086 | 0.45388 | 0.75313 | 4 |
| Ras signaling pathway | 0.18018 | 0.90760 | -0.92308 | -1.22459 | 1 |
| Retrograde endocannabinoid signaling | 0.57762 | 0.90760 | 0.72308 | 0.95790 | 1 |
| Selenocompound metabolism | 0.70293 | 0.93086 | -0.66154 | -0.87762 | 1 |
| Spinocerebellar ataxia | 0.57762 | 0.90760 | 0.72308 | 0.95790 | 1 |
| Starch and sucrose metabolism | 0.74469 | 0.93086 | 0.45332 | 0.75220 | 2 |
| Taste transduction | 0.03895 | 0.90760 | 0.96520 | 1.42851 | 2 |
| Taurine and hypotaurine metabolism | 0.20688 | 0.90760 | -0.90769 | -1.20418 | 1 |
| Tryptophan metabolism | 0.70259 | 0.93086 | 0.66154 | 0.87637 | 1 |
| Tyrosine metabolism | 0.29025 | 0.90760 | 0.73005 | 1.21138 | 4 |
| Ubiquinone and other terpenoid-quinone biosynthesis | 0.21376 | 0.90760 | 0.90769 | 1.20247 | 1 |
| Valine, leucine and isoleucine biosynthesis | 0.27107 | 0.90760 | 0.74157 | 1.23049 | 4 |
| Valine, leucine and isoleucine degradation | 0.24389 | 0.90760 | 0.75431 | 1.25163 | 4 |
| beta-Alanine metabolism | 0.60575 | 0.90760 | -0.58560 | -0.94742 | 4 |
| cAMP signaling pathway | 0.91394 | 0.96679 | -0.41436 | -0.63202 | 3 |
| mTOR signaling pathway | 0.41881 | 0.90760 | -0.80000 | -1.06131 | 1 |

**Supplementary Table 11:** The results of metabolic set enrichment analysis based on longitudinal metabolic profile of severe TBI.

| **Pathway** | **P.Value** | **adj.P.Val** | **ES** | **NES** | **Observed_Hits** |
| --- | --- | --- | --- | --- | --- |
| 2-Oxocarboxylic acid metabolism | 0.9631 | 1.0000 | -0.2681 | -0.5489 | 6 |
| Alanine, aspartate and glutamate metabolism | 0.2942 | 0.7145 | 0.4889 | 1.1472 | 10 |
| Amino sugar and nucleotide sugar metabolism | 0.4968 | 0.7905 | 0.6875 | 1.0371 | 2 |
| Aminoacyl-tRNA biosynthesis | 0.0445 | 0.7145 | -0.5702 | -1.5269 | 14 |
| Arginine and proline metabolism | 0.0864 | 0.7145 | 0.7907 | 1.4366 | 4 |
| Arginine biosynthesis | 0.5627 | 0.8157 | 0.4796 | 0.9282 | 5 |
| Ascorbate and aldarate metabolism | 0.4340 | 0.7905 | 0.7188 | 1.0842 | 2 |
| Biosynthesis of amino acids | 0.1098 | 0.7145 | -0.5144 | -1.3776 | 14 |
| Biosynthesis of cofactors | 0.6964 | 0.8566 | -0.3165 | -0.8287 | 13 |
| Biosynthesis of nucleotide sugars | 0.3164 | 0.7145 | 0.6984 | 1.1659 | 3 |
| Butanoate metabolism | 0.4048 | 0.7905 | 0.5861 | 1.0649 | 4 |
| Carbon metabolism | 0.5435 | 0.8095 | -0.3730 | -0.9284 | 11 |
| Circadian entrainment | 0.3035 | 0.7145 | 0.8615 | 1.1439 | 1 |
| Citrate cycle (TCA cycle) | 0.1041 | 0.7145 | 0.7740 | 1.4063 | 4 |
| Cocaine addiction | 0.3035 | 0.7145 | 0.8615 | 1.1439 | 1 |
| Cysteine and methionine metabolism | 0.6602 | 0.8403 | 0.5781 | 0.8721 | 2 |
| Ether lipid metabolism | 0.0477 | 0.7145 | 0.9846 | 1.3073 | 1 |
| Folate biosynthesis | 0.4275 | 0.7905 | 0.8000 | 1.0622 | 1 |
| FoxO signaling pathway | 0.3035 | 0.7145 | 0.8615 | 1.1439 | 1 |
| GABAergic synapse | 1.0000 | 1.0000 | 0.5077 | 0.6741 | 1 |
| Galactose metabolism | 0.1485 | 0.7145 | 0.6889 | 1.3333 | 5 |
| Gastric acid secretion | 0.8757 | 0.9730 | -0.5692 | -0.7544 | 1 |
| Glutamatergic synapse | 0.9403 | 1.0000 | 0.3744 | 0.5648 | 2 |
| Glutathione metabolism | 0.4907 | 0.7905 | -0.6160 | -1.0134 | 3 |
| Glycerolipid metabolism | 0.7855 | 0.8965 | 0.6154 | 0.8171 | 1 |
| Glycerophospholipid metabolism | 0.6059 | 0.8157 | 0.4913 | 0.8927 | 4 |
| Glycine, serine and threonine metabolism | 0.5833 | 0.8157 | -0.4848 | -0.9312 | 5 |
| Glycolysis / Gluconeogenesis | 0.7173 | 0.8634 | -0.4657 | -0.8335 | 4 |
| Glyoxylate and dicarboxylate metabolism | 0.4415 | 0.7905 | -0.4762 | -1.0291 | 7 |
| Histidine metabolism | 0.9875 | 1.0000 | -0.2283 | -0.4673 | 6 |
| Huntington disease | 0.3035 | 0.7145 | 0.8615 | 1.1439 | 1 |
| Inflammatory mediator regulation of TRP channels | 0.7655 | 0.8931 | 0.5052 | 0.7621 | 2 |
| Inositol phosphate metabolism | 0.6059 | 0.8157 | 0.7077 | 0.9397 | 1 |
| Long-term potentiation | 0.3035 | 0.7145 | 0.8615 | 1.1439 | 1 |
| Longevity regulating pathway | 0.1237 | 0.7145 | -0.9538 | -1.2642 | 1 |
| Melanogenesis | 0.5194 | 0.7905 | -0.7538 | -0.9991 | 1 |
| Metabolic pathways | 0.7940 | 0.8965 | -0.2570 | -0.8034 | 51 |
| Morphine addiction | 1.0000 | 1.0000 | 0.5077 | 0.6741 | 1 |
| Nicotinate and nicotinamide metabolism | 0.1209 | 0.7145 | -0.8600 | -1.2750 | 2 |
| Nitrogen metabolism | 0.9403 | 1.0000 | 0.3744 | 0.5648 | 2 |
| Pantothenate and CoA biosynthesis | 0.2811 | 0.7145 | -0.6507 | -1.1645 | 4 |
| Pathways in cancer | 0.1161 | 0.7145 | 0.9538 | 1.2665 | 1 |
| Pathways of neurodegeneration - multiple diseases | 0.3035 | 0.7145 | 0.8615 | 1.1439 | 1 |
| Pentose and glucuronate interconversions | 0.5147 | 0.7905 | 0.7538 | 1.0009 | 1 |
| Phenylalanine metabolism | 0.5134 | 0.7905 | -0.6719 | -0.9961 | 2 |
| Phenylalanine, tyrosine and tryptophan biosynthesis | 0.5134 | 0.7905 | -0.6719 | -0.9961 | 2 |
| Phosphatidylinositol signaling system | 0.6059 | 0.8157 | 0.7077 | 0.9397 | 1 |
| Phospholipase D signaling pathway | 0.3035 | 0.7145 | 0.8615 | 1.1439 | 1 |
| Platelet activation | 0.6350 | 0.8301 | 0.6923 | 0.9192 | 1 |
| Porphyrin and chlorophyll metabolism | 0.9665 | 1.0000 | 0.3439 | 0.5188 | 2 |
| Primary bile acid biosynthesis | 0.1525 | 0.7145 | -0.8438 | -1.2509 | 2 |
| Propanoate metabolism | 0.6975 | 0.8566 | 0.6615 | 0.8784 | 1 |
| Purine metabolism | 0.2500 | 0.7145 | 0.5073 | 1.1902 | 10 |
| Pyrimidine metabolism | 0.2086 | 0.7145 | -0.8125 | -1.2046 | 2 |
| Pyruvate metabolism | 0.3152 | 0.7145 | 0.6309 | 1.1463 | 4 |
| Ras signaling pathway | 0.4275 | 0.7905 | 0.8000 | 1.0622 | 1 |
| Retrograde endocannabinoid signaling | 0.3035 | 0.7145 | 0.8615 | 1.1439 | 1 |
| Selenocompound metabolism | 0.0983 | 0.7145 | -0.9692 | -1.2846 | 1 |
| Spinocerebellar ataxia | 0.3035 | 0.7145 | 0.8615 | 1.1439 | 1 |
| Starch and sucrose metabolism | 0.3362 | 0.7354 | 0.7656 | 1.1549 | 2 |
| Taste transduction | 0.1302 | 0.7145 | 0.8750 | 1.3199 | 2 |
| Taurine and hypotaurine metabolism | 0.1542 | 0.7145 | -0.9385 | -1.2438 | 1 |
| Tryptophan metabolism | 0.7277 | 0.8634 | -0.6462 | -0.8564 | 1 |
| Tyrosine metabolism | 0.2612 | 0.7145 | 0.6616 | 1.2022 | 4 |
| Ubiquinone and other terpenoid-quinone biosynthesis | 0.5194 | 0.7905 | -0.7538 | -0.9991 | 1 |
| Valine, leucine and isoleucine biosynthesis | 0.2984 | 0.7145 | -0.6452 | -1.1546 | 4 |
| Valine, leucine and isoleucine degradation | 0.4544 | 0.7905 | -0.5796 | -1.0373 | 4 |
| beta-Alanine metabolism | 0.4568 | 0.7905 | -0.5785 | -1.0353 | 4 |
| cAMP signaling pathway | 0.6404 | 0.8301 | 0.5238 | 0.8744 | 3 |
| mTOR signaling pathway | 0.1237 | 0.7145 | -0.9538 | -1.2642 | 1 |


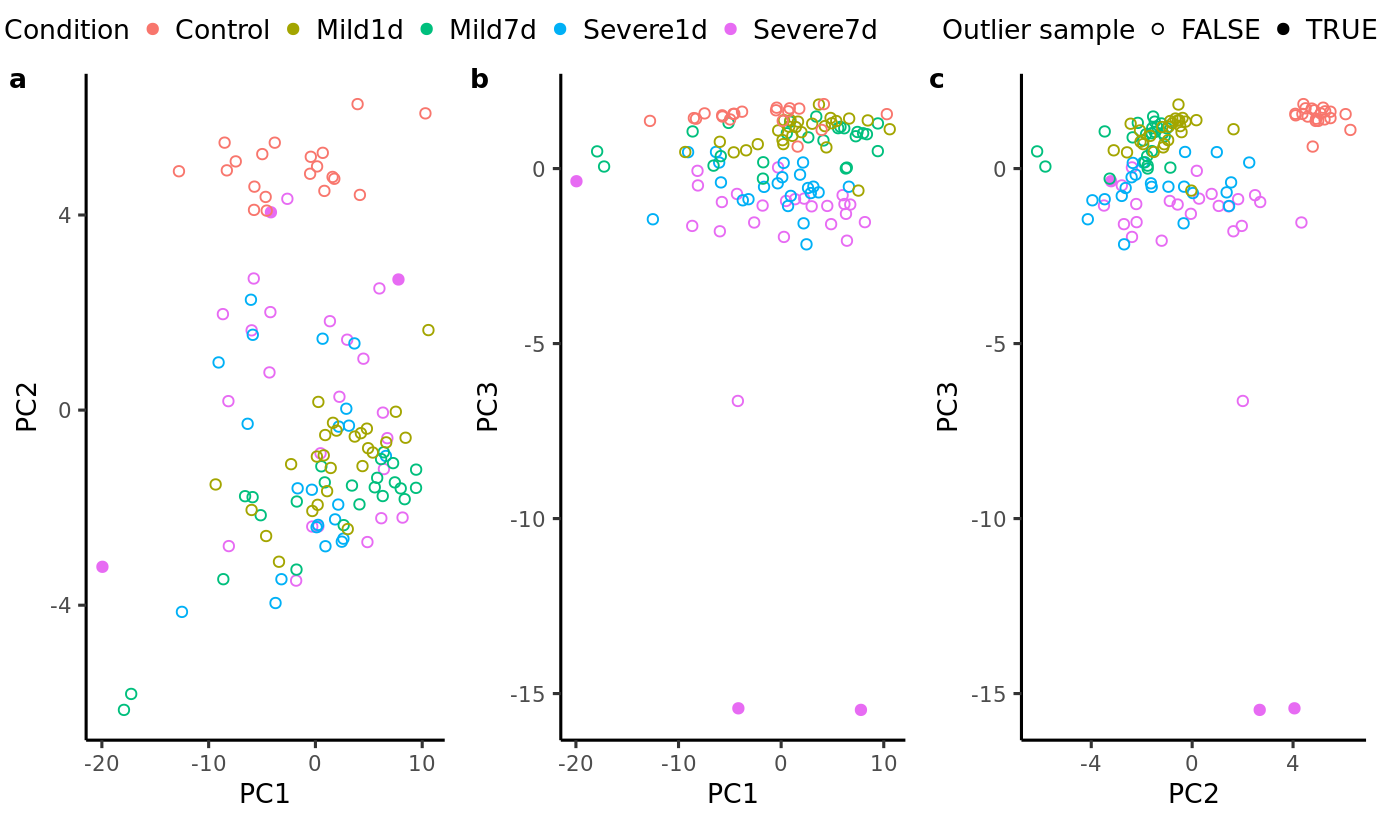


**Supplementary Figure 1**: Principal Component Analysis (PCA) of metabolomics data to determine the existence of systematic variation or outlier.


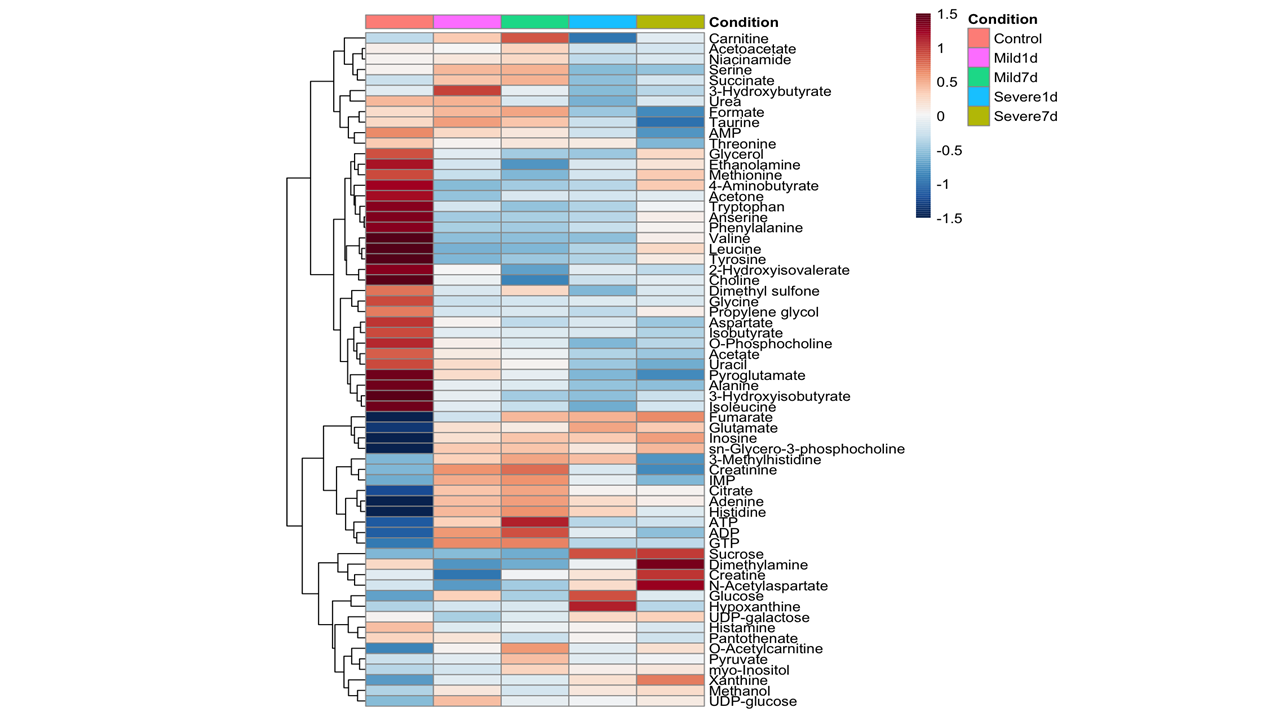


**Supplementary Figure 2**: Heatmap showing change in the concentration of metabolite values over time and trauma. While differentially high abundant metabolite shown in red, less abundant metabolites has shown in blue.


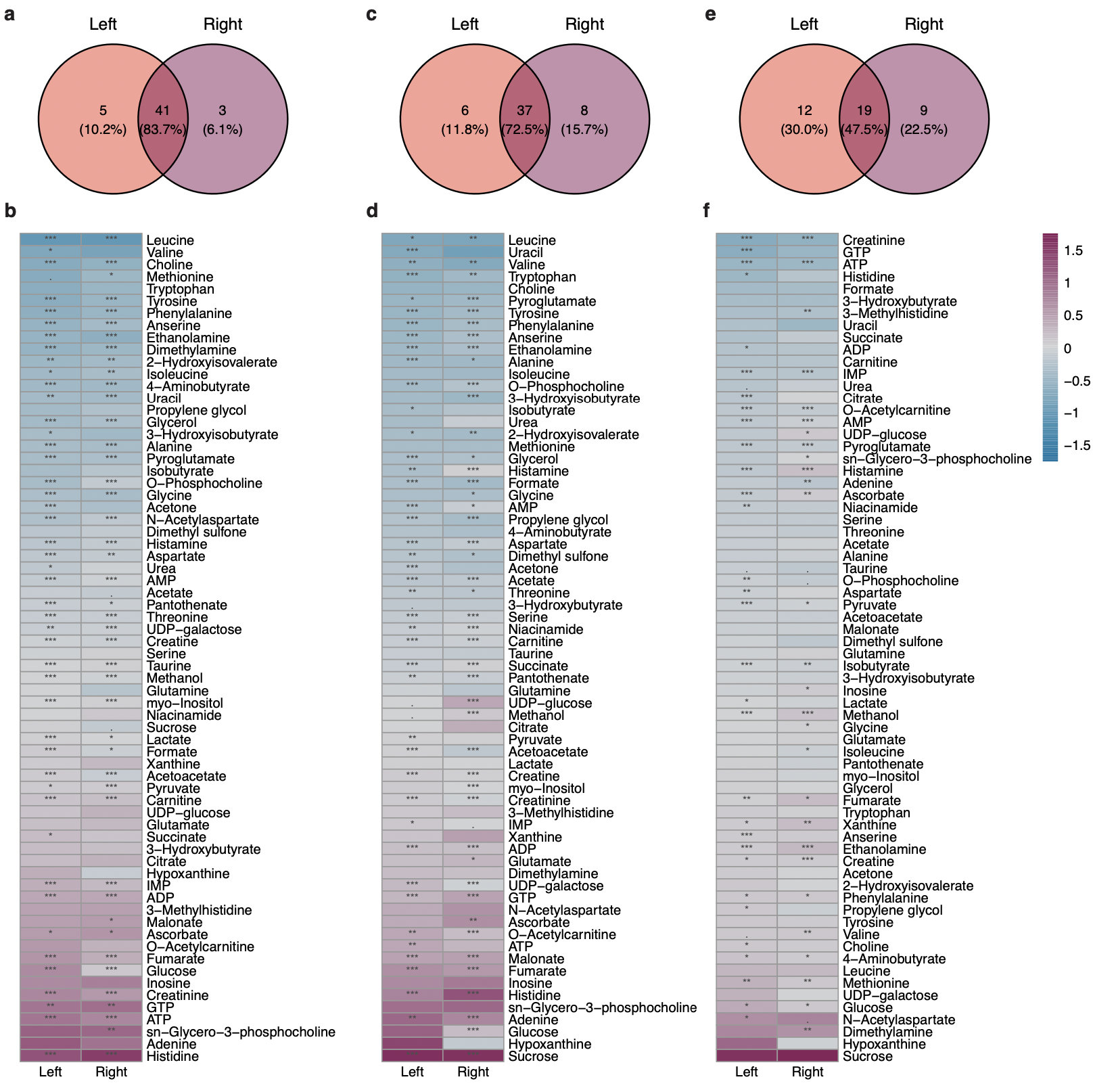


**Supplementary Figure 3**: Venn diagrams and heatmap indicating similarity of the metabolic profile change between left and right hemispheres of the mouse models when all severe TBI compared with healthy controls **(A-B)**, when all mild cases compared with healthy controls **(C-D)**, and when all severe TBI cases were compared with all mild TBI cases **(E-F)**, respectively.

**
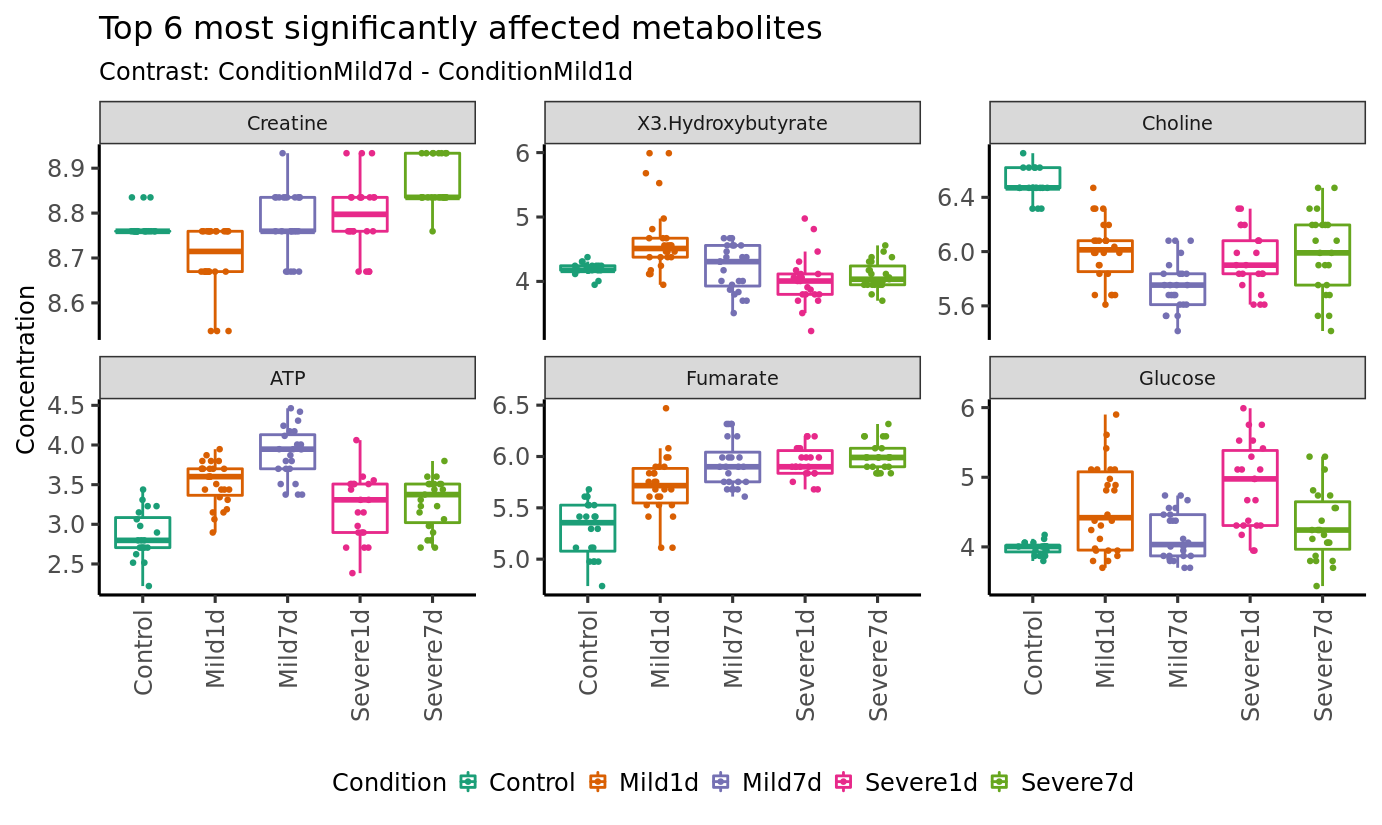
**

**Supplementary Figure 4**: Boxplots of top six most significantly affected metabolites comparing initial injury of Mild TBI (24 hours) and Mild TBI post-injury (7 days).


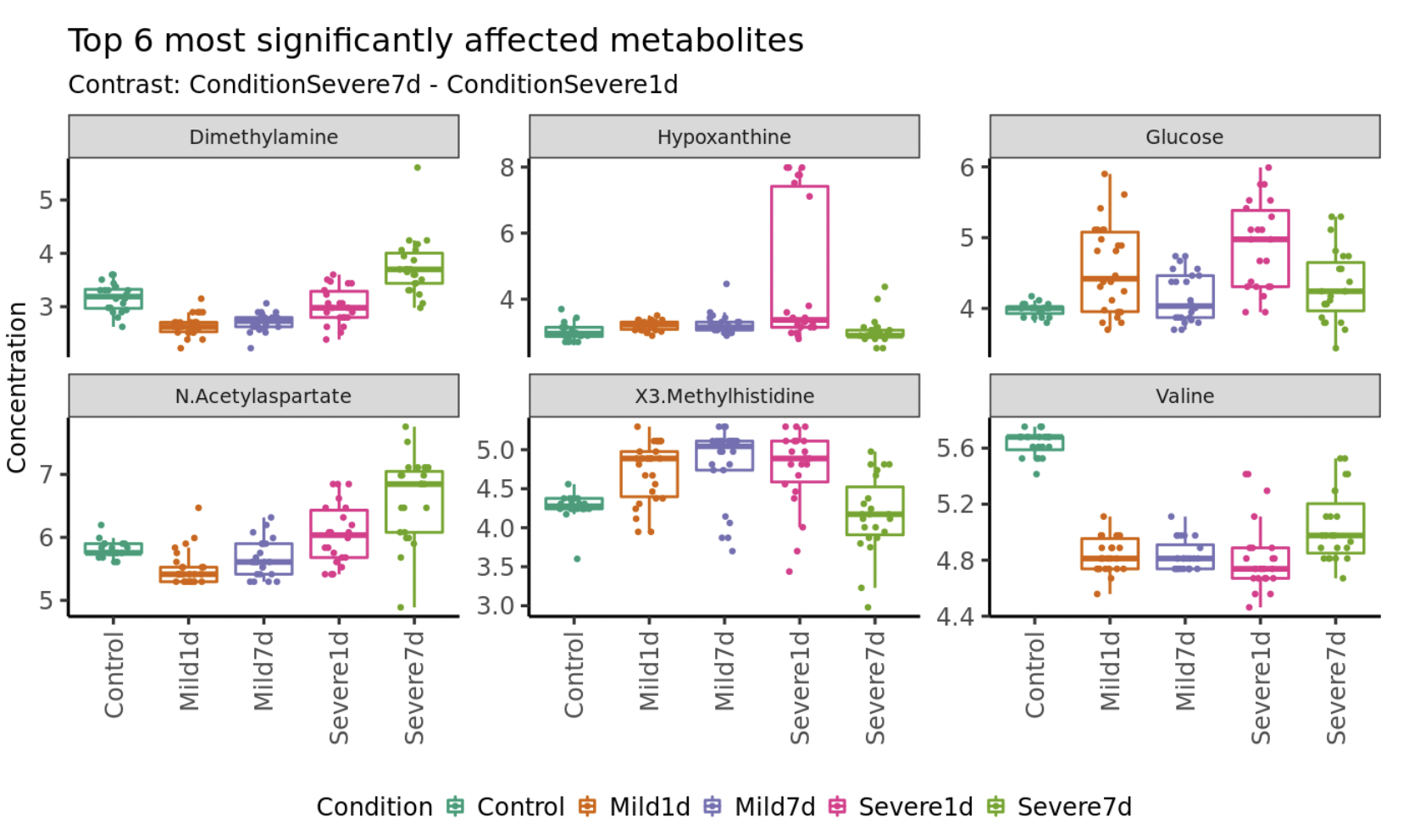


**Supplementary Figure 5**: Linear model indicating top six most significantly affected metabolites when the initial injury of severe TBI (24 hours) and post-injury (7days) were compared.


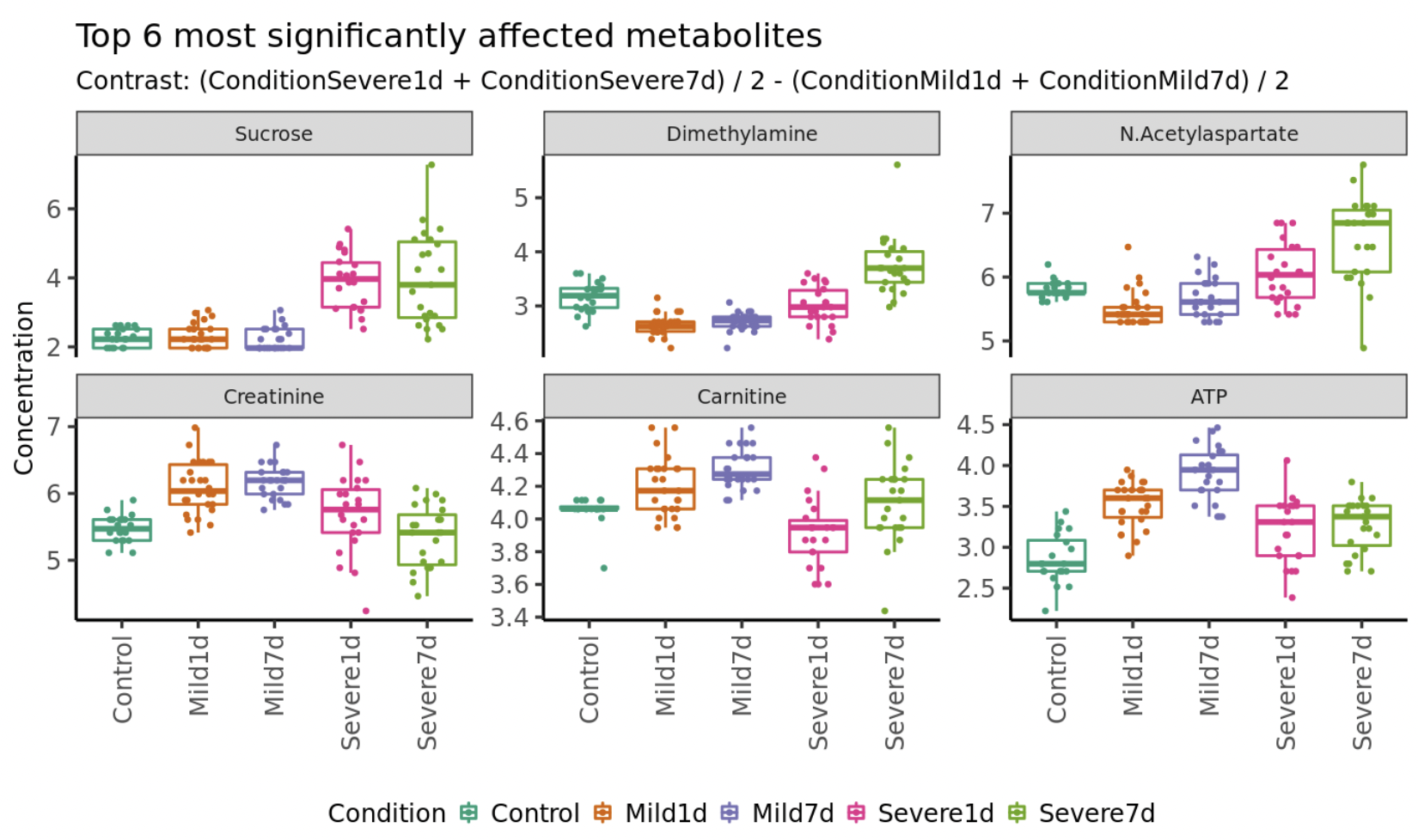


**Supplementary Figure 6**: Linear model indicating top six most significantly affected metabolites when all mild TBI (24 hours and 7 days) cases compared with all severe TBI (24 hours and 7 days).


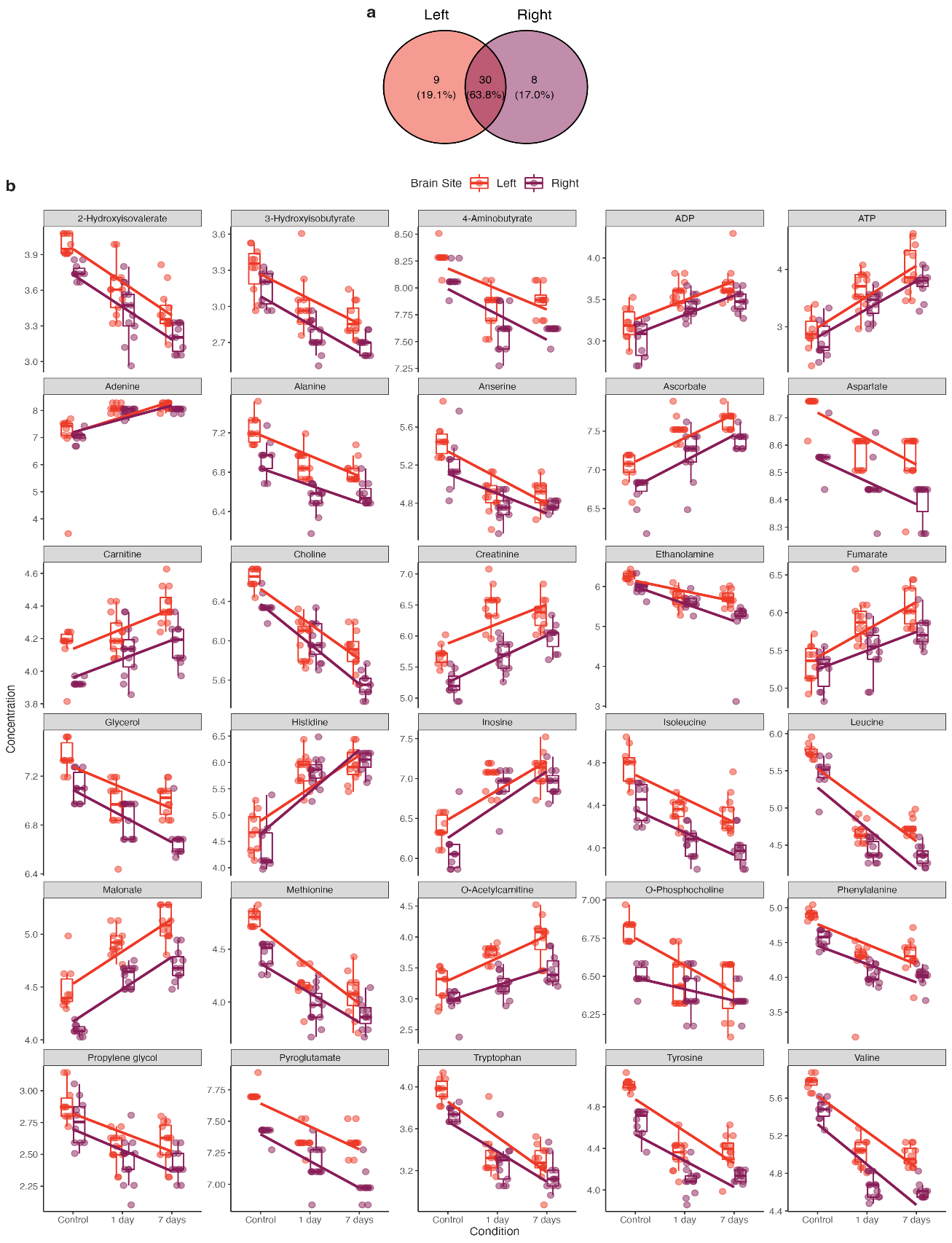


**Supplementary Figure 7**: **(a)** Venn diagram indicating similarity of the longitudinal metabolic profile change between left and right hemispheres of the mouse models of mild TBI and **(b)** change of the metabolites significantly affected in both hemispheres.


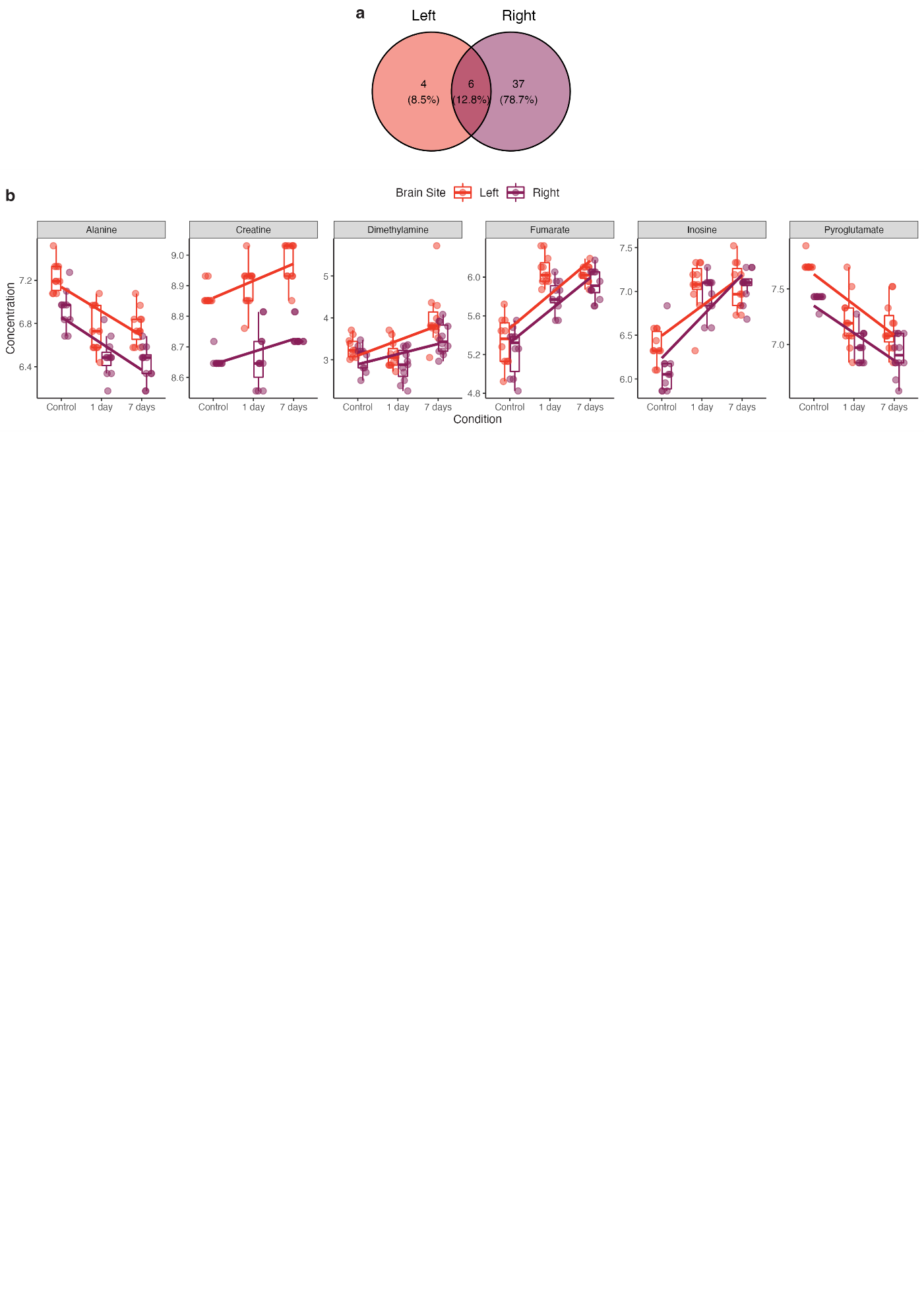
**Supplementary Figure 8**: **(a)** Venn diagram indicating similarity of the longitudinal metabolic profile change between left and right hemispheres of the mouse models of severe TBI and **(b)** change of the metabolites significantly affected in both hemispheres.


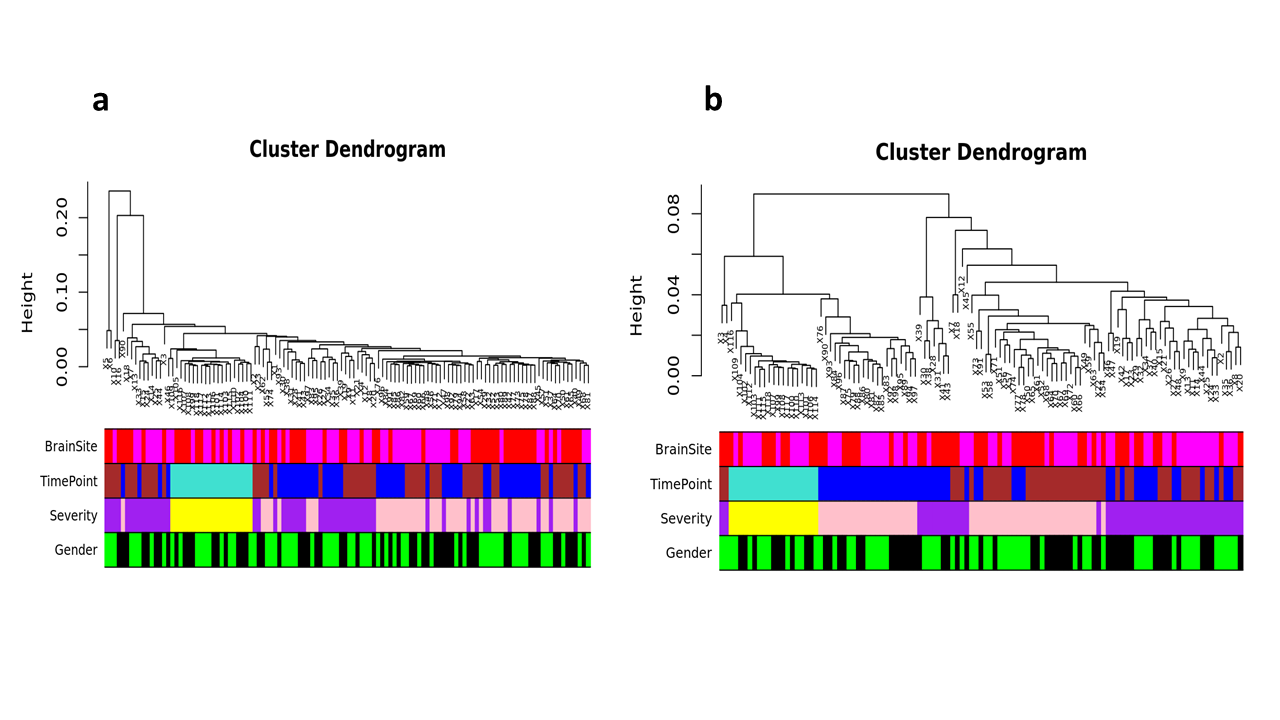


**Supplementary Figure 9**: Cluster analysis of data **(a)** before and **(b)** after outlier detection
